# Nanoengineering of N-doped Mesoporous Carbon Nanoparticles with Adjustable Internal Cavities via Emulsion-Induced Assembly

**DOI:** 10.3390/ma15072591

**Published:** 2022-04-01

**Authors:** Cong Wang, Xiaoxi Zhao, Xiufang Wang, Yong Tian

**Affiliations:** School of Pharmacy, Guangdong Pharmaceutical University, Guangzhou 510006, China; wangcong199662@163.com (C.W.); zhaoxx_17@163.com (X.Z.)

**Keywords:** N-doped, carbon materials, porous materials, controllable synthesis, nanoparticles

## Abstract

The preparation of mesoporous carbonaceous materials with particularly adjustable morphology is currently a hot area of research in mesoporous materials. Herein, a novel approach is reported for the construction of N-doped multicavity mesoporous carbon nanoparticles (NMMCNs) based on the “emulsion swelling–acid curing mechanism” using a nanoemulsion assembly method under a high-speed shearing force. Intriguingly, this approach adopted a novel acid (HCl) curing procedure. Impressively, the morphology evolution from an internal multicavity to a single cavity and then to a non-cavity interior structure could be accomplished by simply varying the synthesis parameters. Additionally, this synthesis approach ingeniously overcame the following problems: (i) technically, the employment of high temperatures and high pressures in traditional hydrothermal reaction curing environments is avoided; (ii) this approach removes the requirement for silicon coating, which provides a limited pyrolysis condition, to obtain a multi-chamber structure. Resveratrol (Res) is an insoluble natural medicine and was successfully loaded into NMMCNs, thereby the Res–NMMCNs delivery system was constructed. Importantly, the Res–NMMCNs delivery system could still retain the antitumor and antioxidant activity of Res in vitro.

## 1. Introduction

For many years, mesoporous carbon nanoparticles (MCNs) have played an irreplaceable role in catalysis, energy storage conversion, adsorption separation, drug storage and release and other fields due to their large specific surface area, large pore volume, unique construction and other fantabulous physicochemical properties [1,2,3,4,5,6,7,8]. In fact, the performance of MCNs is unquestionably affected by their structural and morphological characteristics, such as internal hollow chambers, the thickness of the shell, pore size distribution and even the surface roughness of the particle. Accordingly, the preparation of MCNs with specific particle morphological configurations has always been one of the hot issues in carbon nanomaterial research.

Up to now, there have been various ways to synthesize mesoporous materials: the hard-templating method [9,10], the evaporation-induced self-assembly method (EISA) [11,12], the aqueous solution self-assembly method [13,14], the hydrothermal method [8,15], etc. The preparation of MCNs in numerous morphologies has been achieved through various mediums. For the preparation of MCNs, the hard-templating method was introduced first. However, it is arduous to achieve industrial production due to the complicated etching process and the fact that the morphology and mesoporous aperture of the material strongly depended on the master template. Therefore, it is hard to realize adjustable morphology and structural control by varying the ratio of raw materials, which makes the method subject to serious limitations. For example, Zhang et al. [16] employed rigid particle silica as the core templates to prepare hollow MCNs. In their method, the hollow structure and shell thickness were highly determined by the silica template and the shell was relatively thin, resulting in the generation of numerous broken hollow MCNs. Additionally, the aqueous self-assembly and hydrothermal methods are ordinarily the most commonly utilized strategies for obtaining MCNs. They have become popular choices among scientists because they do not necessitate complex operation processes, such as template removal or the usage of a large amount of volatile chemical solvents. For instance, Liu et al. [17] presented a dual-template approach to the aqueous solution at 80 °C to prepare the N-doped MCNs with rough surfaces. At the same time, Wang’s group [8] prepared small mesoporous hydrothermal carbon nanospheres using the hydrothermal approach at 140 °C, which is applied in the field of biomedical research. Although the MCNs that were prepared by predecessors have played an active role in multiple research fields, the small particle size MCNs with adjustable internal chambers are still in the exploratory stage. Multicavity mesoporous carbon with a small particle size may be more conducive to the realization of drug loading and could also be employed as a carrier material for further application. Delightfully, the emulsion-induced method that has been developed in recent years has become a research hotspot for the preparation of various mesoporous carbon materials with different morphologies and anisotropies. The emulsion-induced approach provides the benefits of the easy removal of templates, large-scale production and environmental friendliness. In comparison to the other approaches, the emulsion-induced method, which is founded on the self-assembly of the precursor molecules and the different emulsion droplets systems as the core templates, is more flexible. It provides an opportunity to construct multi-morphology, multi-chamber and highly interconnected apertures, which can vastly improve the mass transportation of the drug carrier. For example, Zhang and his colleagues [18] reported an approach using surface-free energy-induced assembly to synthesize multicavity carbon microspheres. Multicavity carbon microspheres were eventually acquired by employing a small nanoemulsion with a high uniformity (triolein droplets) as the main building block and a coating of silica to a confined pyrolysis condition. Furthermore, Chen et al. [19] prepared a flask-like hollow carbonaceous material by utilizing sodium oleate as the precursor of the emulsion to produce oleic acid under acidic conditions and formed an emulsion with water and surfactant P123. Likewise, by employing F127 as the surfactant, oil phase 1,3,5-trimethyl-benzen (TMB) as the emulsion template and dopamine as the carbon source, an emulsion-induced interface assembly strategy for the preparation of MCNs with diverse structures (such as vesicles, walnut-like and dendritic-like MCNs) was demonstrated by Yang et al. [20]. Even though intensive efforts have been devoted to fabricating MCNs with distinct various morphologies and structures using the emulsion-induced technique, some issues within the synthesis process still exist: (1) technically, the technique adopts a hydrothermal high-temperature reaction and solidifies in a high-temperature and high-pressure environment and so, correspondingly, the Teflon-lined stainless steel autoclave equipment that is utilized also has certain safety hazards; (2) in terms of synthesis, it is difficult to remove the requirement for a silicon coating to provide a limited pyrolysis condition and obtain a multi-chamber structure, which undoubtedly increases the cumbersome experimental process; (3) by comparison, the as-synthesized MCNs manifest large particle diameters and smooth surfaces.

Throughout the development trends of the synthesis of MCNs, the excellent characteristics of mesoporous carbon materials have evolved from an inherent single morphology and small mesoporous aperture to the synergistic effect of a unique morphology, small particle size, large pore size and rigid framework; thus, gradually realizing the improved application performance of MCNs as catalyst carriers, adsorption separators, drug carrier materials, etc. Even though extensive research has been conducted on the preparation of MCNs with multi-morphology and various structures [21,22,23], certain problems still exist, such as the utilization of high-temperature and high-pressure hydrothermal reactors and complicated silicon coating steps. Furthermore, in an exceedingly simple oil–water microemulsion reaction system, the evolution of MCNs with distinctly different internal chamber structures remains troublesome to attain. Thus, it is still a challenge to explore methods for obtaining MCNs with stable thermosetting properties under low-temperature curing conditions, adjustable particle sizes, rough surfaces and inter-changeable internal structures.

Herein, this research utilized a high-speed shearing force to create an emulsion that self-assembled with polybenzoxazine resin in an aqueous solution to prepare NMMCNs with cherimoya-shaped rough surfaces and changeable internal cavities under acid curing conditions. Moreover, the “emulsion swelling–acid curing mechanism” was also proposed to explain their growth mechanism. This formation approach not only avoided the high-temperature curing process and the employment of autoclave equipment, but also further supplemented the swelling mechanism of the emulsion strategy. Benzoxazines are an interesting class of monomers, which are produced by the Mannich condensation reaction of organic primary amine, phenol derivatives and formaldehyde [24,25,26,27]. Owing to its delicate molecular design flexibility, the content and type of nitrogen functional groups in benzoxazine could be simply regulated by altering the type or ratio of primary amines. The obtained NMMCNs had excellent characteristics, such as high nitrogen doping contents, small particle sizes, large apertures, unique and adjustable morphologies and hierarchical mesoporous structures. On the other hand, cytotoxicity and cell uptake assays were conducted to evaluate the biocompatibility of the NMMCNs. In the preliminary investigation of the in vitro release performance of NMMCNs as drug carriers loaded with Res, it was found that NMMSNs could significantly increase the release of Res, which provides a powerful arsenal for the application of MCNs in drug carriers. Moreover, the increase in N-doped content considerably improves the wettability of carbon materials [17,28,29] and the development of this capability could further promote their application performance as drug carriers.

## 2. Experimental Sections

### 2.1. Chemicals and Materials

The F127 (PEO–PPO–PEO) was purchased from BASF. The diethyl phthalate (DEP, 99.5%), 3-aminophenol (3-AP, 98%), formaldehyde (F, 37%), ethanol, ethylenediamine (EDA), hydrochloric acid (HCl, 37%) and Resveratrol (Res, 99%) were purchased from the Guangzhou Chemical Company (Guangzhou, China). The 2,2-Diphenyl-1-picrylhydrazyl (DPPH•), fluorescein isothiocyanate (FITC), dimethyl sulfoxide (DMSO) and 3-(4, 5-dimethyl-2-thiazolyl)-2, 5-diphenyl-2-H-tetrazolium bromide (MTT) were purchased from Sigma (St. Louis, MO, USA). Deionized water was used for all experimental water. All chemicals were used in their original state without further purification.

### 2.2. Synthesis of NMMCNs with Cherimoya-Shaped Rough Surfaces

In the typical synthesis strategy, 0.4 g of F127 was dissolved in a mixture solution containing 5 mL of ethanol and 35 mL of deionized water under magnetic stirring at 40 °C. Subsequently, DEP was then dissolved in the above solution and agitated for 30 min at 600 rpm to create an oil-in-water primary emulsion. After that, the temperature was quickly lowered to 20 °C and a homogenizer was employed to prepare the secondary emulsion under a high-speed shearing force of 16,000 rpm. After 10 min, 0.25 mL of formaldehyde solution, 0.1 mL of EDA and 0.3 g of 3-AP were added and mixed for another 60 min. The reaction temperature was then elevated to 50 °C for 22 h and the pH value of the reaction solution was adjusted to around 5 with 2 M of HCl solution before continuing the reaction for another 10 h. Finally, the reaction solution was centrifuged and the resulting colloidal precipitate was washed three times with deionized water before being dried fully at 50 °C to yield polybenzoxazine (PBZ) resin. Under the protection of an N_2_ environment, the PBZ resin was carbonized at 350 °C for 3 h and then at 600 °C for another 3 h at a heating rate of 2 °C/min. The obtained black powder samples were labeled as NMMCNs-x, with x representing the sample number of the different synthesis parameters. The specific experimental parameters are presented in Table 1.

### 2.3. Cytotoxicity Assays of NMMCNs-2 with Cherimoya-Shaped Rough Surfaces

The cytotoxicity of NMMCNs-2 was evaluated by an MTT assay using 3T3 cells as cell line models. The cells were seeded in 96-well plates with a density of 1 × 10^4^ cells in each well and incubated in a constant-temperature carbon dioxide incubator at 37 °C and 5% CO_2_. When the cells grew to 80%, the materials that were dispersed in the complete medium were added according to the designed concentration gradient of 0, 6.25, 12.5, 25, 50, 100 or 200 μg/mL. The medium was withdrawn after 24 h of incubation and the cells were washed three times with fresh PBS. To generate formazan crystals, 20 μL of 5 mg/mL MTT solution was supplemented into each well and incubated with 3T3 cells at 37 °C for 4 h. The obtained formazan was then dissolved in 200 μL of DMSO and the cells were shaken slowly for 10 min. The microplate reader (BioTek, Winooski, VT, USA) was employed to measure the absorbance value at 490 nm [30]. The cell survival rate was determined according to the following formula:

Cell Viability (%) = (mean optical density of the experimental group)/(mean optical density of control group) × 100%

### 2.4. Hemolysis Experiment

In order to test the blood biocompatibility of NMMCNS-2, blood samples were collected from the abdominal aorta of mice. Red blood cells (RBC) were collected by centrifugation at 4 °C and 3500 rpm for 10 min and were then washed with cold PBS three times until the supernatant was clarified. Then, the RBCs were prepared in a 2% red blood cell suspension. Next, 1 mL of PBS containing different NMMCNs-2 material concentrations (10, 20, 50, 100, 200 or 500 μg/mL) was combined with 1 mL of 2% red blood cell suspension and incubated at 37 °C for 3 h. Meanwhile, 1 mL of PBS was added into 1 mL of 2% red blood cell suspension as the negative control and 1 mL of deionized water was mixed with 1 mL of 2% red blood cell suspension as the positive control. After incubation at 37 °C for 3 h, the samples were centrifuged at 4 °C and 3500 rpm for 10 min. The absorbance value of the supernatant was determined at a wavelength of 541 nm [8,29] by UV-vis spectroscopy (UV-vis 752N). A sample of 500 μg/mL was selected to observe the morphology of the RBCs under the SEM. The hemolysis rate of the sample was calculated as follows (A stands for absorbance):

Hemolysis (%) = ([A]_experiment_ − [A]_positive control_)/([A]_negative control_ − [A]_positive control_) × 100%

### 2.5. Cell Uptake Assay

FITC-grafted NMMCNs-2 was obtained by grafting FITC onto the surface of NMMCNs-2 according to the literature method. Then, 3T3 cells in the logarithmic growth phase were harvested for the experiments. They were cultured in a complete medium after digestion with 0.25% trypsin (HyClone). After complete digestion, the isolated cell suspension was centrifuged and reconstituted with an equal fresh medium containing FITC-grafted NMMCNs-2 (100 μg/mL). After incubating for 1, 2, 4 and 12 h, the cells were fixed in 4% paraformaldehyde for 10 min and washed three times with a serum-free medium. Then, the cells were washed three times with a PBS buffer and further stained with 10 μg/mL of Hochest 33,258 for 10 min, followed by imaging with a confocal microscope (LSM700, Zeiss, Jena, Germany).

### 2.6. Construction of the Res-Loaded Delivery System and In Vitro Release Profile Study

Res was chosen as the model drug and Res was loaded into NMMCNs-2 via the solvent evaporation method to construct the Res-loaded delivery system. Then, 40 mg of NMMCNs-2 was added to 10 mL of a Res–methanol mixed solution with a Res concentration of 4 mg/mL. To guarantee the best drug loading efficiency, the resultant mixture was swirled at an adequate speed for 30 h in a closed and dark environment at room temperature. Finally, the methanol solvent was allowed to evaporate for nearly 20 h at 50 °C, which fully obliterated the methanol solvent. The Res-loaded sample was denoted as Res–NMMCNs-2-(1:2). Notably, 1:2 represents the mass ratio of Res to NMMCNs-2. The Res-loaded delivery systems of Res–NMMCNs-2-(2:1), Res–NMMCNs-2-(1:1) and Res–NMMCNs-2-(2:3) were constructed by changing the dosage of NMMCNs-2. These Res-loaded samples were collectively termed Res–NMMCNs-2.

The drug loading was determined by extracting 10 mg of Res–NMMCNs-2 with 25 mL of methanol under ultrasonic conditions. After ultrasonic treatment for 15 min and stirring for 18 h, the Res was completely released from the mesoporous channel of NMMCNs-2. Then, the mixture was filtered with a 0.22 μm filter to obtain a supernatant. Subsequently, the drug content was measured with UV-vis at a wavelength of 302 nm. The drug loading (DL) was then calculated according to the following formula:

DL (%) = (W_0_/W) × 100%

where W_0_ refers to the mass of Res in Res–NMMCNs-2 and W refers to the total mass of Res–NMMCNs-2.

The release experiments of Res from the Res–NMMCNs-2 material were performed under four different conditions, which simulated gastric juice (HCl solution of pH 1.2), intestinal juice (PBS of pH 5.8), saliva (PBS of pH 6.8) and body fluid (PBS of pH 7.4). Firstly, Res–NMMCNs samples that were equivalent to 10 mg of Res were dispersed in buffers of different pH values and placed in dialysis bags (molecular weight cut-off of 3500). Then, the dialysis bags were immersed in 150 mL of different dissolved media and oscillated at 120 rpm. To simulate in vivo release characteristics, 5 mL of release medium was extracted at an appropriate sampling time, followed by the addition of the same amount of fresh medium. After filtering and appropriately diluting the extract, the absorbance was determined by UV-vis spectrophotometry at 302 nm to detect the concentration of Res in the released solution. For comparison, a control experiment was carried out with pure Res instead of Res–NMMCNs-2 under the same conditions. Furthermore, the release results were fitted to five mathematical kinetic models: zero order, first order, Higuchi model, Weibull model and Ritger–Peppas model.

### 2.7. Determination of DPPH• Radical Scavenging Activity of the Res–NMMCNs-2 Delivery System

The antioxidant capacity of Res–NMMCNs-2 is based on its ability to scavenge DPPH•. So, 2 mL of different concentrations of Res–NMMCNs-2 (dissolved in ethanol) were added to 2 mL of DPPH• ethanol solution (0.1 mg/mL). Subsequently, the mixed solution was then thoroughly mixed and incubated at room temperature in the dark for 1 h and the absorbance at 517 nm [31] was recorded. A blank reference without any Res–NMMCNs-2 acted as the control. Finally, the percentage of DPPH• scavenging was calculated according to the following formula (A stands for absorbance):

DPPH• scavenging rate (%) = (1 − A_sample_/A_control_) × 100%

An ethanol solution of NMMCNs-2 with the same concentration was employed as the comparative sample to determine antioxidant capacity. The experimental procedure was the same as above.

### 2.8. In Vitro Antitumor Activity Test of the Res–NMMCNs-2 Delivery System

The in vitro antitumor activity of Res–NMMCNs-2 was evaluated using mouse cancer cells (Hepa1-6) as the tumor cell models. The same experimental procedure was followed as described in Section 2.3.

### 2.9. Characterization

Transmission electron microscope (TEM) measurements were conducted on an FEI-Talos F200X (Thermo Fisher Scientific, Waltham, MA, USA), which was operated at 10 kV. Scanning electron microscopy (SEM) images were collected on a Zeiss Sigma 300 (Jena, Germany) field emission electron microscope. Dynamic light scattering (DLS) was conducted at 25 °C on a ZS Nano S Instrument (Malvern. Inc., Worcestershire, UK). The N_2_ sorption isotherms were performed on a Micromeritics Tristar 3020 (USA) at −196 °C. The sample powders were degassed at 200 °C in a vacuum for 6 h before measurement. The specific surface areas were calculated by employing the Brunauer–Emmett–Teller (BET) method. Correspondingly, the pore size distribution (PSD) and pore volume were calculated from the PSD curves in the adsorption branches of the isotherms using the Barrett–Joyner–Halenda (BJH) method. The surface compositions of the NMMCNs obtained with different amounts of EDA were measured by X-ray photoelectron spectroscopy (XPS) and recorded with an ESCALAB 250 X-ray photoelectron spectrometer (USA). Wide-angle X-ray diffraction (XRD) patterns were collected on a PAN Analytical Inc. X’Pert Pro with Cu–Kα radiation (50 kV and 300 mA). Raman spectra was recorded with a microscopic Raman spectrometer (Thermo Fisher DXR 2xi, Waltham, MA, USA). Fourier-transform infrared (FTIR) spectra were recorded on an infrared spectrometer VERTEX 70 (Bruker Inc, Germany). The thermogravimetric (TG) characterization was performed on an STA449C thermogravimetric analyzer in the range of 25–800 °C under N_2_ protection at 20 °C min^−1^. The differential scanning calorimetry (DSC) was conducted on a DSC-4000 (PerkinElmer, Waltham, MA, USA) under the protection of N_2_ from room temperature to 500 °C.

## 3. Results and Discussions

### 3.1. Structural Characterization

Innovatively, MCNs with distinct internal cavities were successfully produced by the self-assembly of benzoxazine resin with an emulsion prepared by a high-speed shearing force, which was based on the “emulsion swelling–acid curing mechanism”. In the synthetic system, a series of NMMCNs-x with cherimoya-shaped rough surfaces and adjustable internal cavities were obtained by employing the amphiphilic surfactant F127 as a template, 3-AP and formaldehyde as carbon sources and DEP as the swelling agent under EDA alkaline conditions. Notably, EDA was not only the primary amine compound involved in the construction of the benzoxazine resin, but it was also a basic catalyst and nitrogen source that could be used to realize the nitrogen doping of MCNs. The successful synthesis of NMMCNs-2 with adjustable internal cavities mainly underwent three essential processes: (i) the preparation of the DEP–F127–H_2_O secondary emulsion with a smaller and uniform particle size; (ii) the self-assembly of DEP–F127–PBZs SSUs; and (iii) the introduction of the acid curing procedure to enhance and strengthen the degree of cross-linking between the DEP–F127–PBZs SSUs.

**Figure 1:**
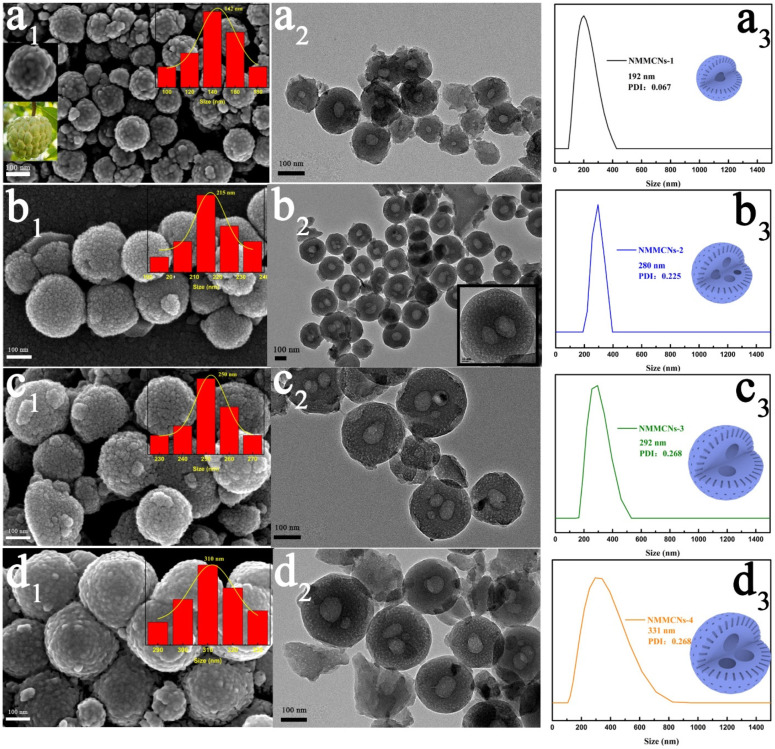
The SEM images (**a_1_**–**d_1_**), TEM images (**a_2_**–**d_2_**) and particle size distributions derived from DLS (**a_3_**–**d_3_**) of NMMCNs synthesized with different DEP contents. (Row (**a**), NMMCNs-1; Row (**b**), NMMCNs-2; Row (**c**), NMMCNs-3; Row (**d**), NMMCNs-4).

In order to evaluate the effect of the oil phase (DEP) on the internal chamber structure of the NMMCNs, a series of NMMCNs were prepared by changing the amount of DEP. When initially trying to employ 0.1 mL of DEP as the nanoemulsion, it could be seen from the SEM image (Figure 1a_1_) that the obtained NMMCNs-1 exhibited a particle size of about 142 nm with a cherimoya-shaped rough surface. The partially magnified SEM image (Figure 1a_1_, upper left inset) provides a clearer view of the surface of the MCNs with many prominences resembling the surface protrusions of cherimoya fruit (Figure 1a_1_, lower left inset), which could have been derived from the accumulation of small F127–DEP–PBZs SSUs. As illustrated in Figure 1a_2_, there was a cavity of about 35 nm inside the NMMCNs-1. With the amount of DEP continuously increasing, the obtained NMMCNs-2, NMMCNs-3 and NMMCNs-4 (Figure 1b_1_–d_1_) all exhibited the same surface structural characteristics (comprising very small and coarse particles) as NMMCNs-1. Surprisingly, the TEM image of NMMCNs-2 (Figure 1b_2_) depicts a multicavity structure with one to four chambers (about 60–72 nm) of different sizes in the case of 0.2 mL of DEP, which can also be distinctly observed in the enlarged TEM image (Figure 1b_2_, inset). The mesopores were obviously visible on the surface of NMMCNs-2 with a particle size of 215 nm. As the quantity of the emulsion was brought to 0.3 and 0.4 mL, it can be observed in Figure 1c_2_,d_2_ that the internal cavity enlarged to 78 nm and 87 nm, respectively, and that the particle size increased from 250 nm to 310 nm. Obviously, for the NMMCNs-1 to NMMCNs-4 samples, the cavity size and particle diameter gradually enlarged with the increase in the dosage of DEP and the internal structure changed from single cavity to multicavity, which also indicates that DEP was the “template of maternal emulsion droplet” that was formed by the cavity structure. The DLS characterization revealed that the average particle diameter of samples NMMCNs-1 to NMMCNs-4 were 192, 280, 292 and 331 nm, respectively. The results of the DLS data (Figure 1a_3_–d_3_) were slightly larger than the particle size of the SEM and TEM images, which could have been aroused by the hydration layer on the surface of the particle [29].

**Figure 2:**
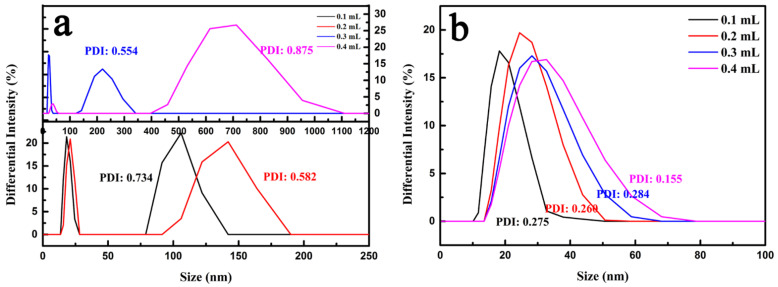
The DLS profiles of the primary emulsion (**a**) and secondary emulsion (**b**).

To further demonstrate the distinction between the internal structures of the particles that was caused by the difference between the amounts of the DEP emulsion droplets, we measured the particle size of both the primary emulsion and the secondary emulsion (prepared by a high-speed shearing force) through a DLS experiment. As displayed in Figure 2a,b, the DLS curves of the primary emulsion and the secondary emulsion were in sharp contrast. The primary emulsion PSD curves exhibited a bimodal distribution with a large polydispersity index (PDI) of 0.734, 0.582, 0.554 and 0.875, respectively. Nevertheless, the particle sizes of the generated secondary emulsion droplets were 20.71, 26.64, 29.62 and 32.48 nm and their PDI value was less than 0.3, meaning that the obtained secondary nanoemulsion had a high degree of uniformity. Therefore, we speculate that the distribution, aggregation and assembly of the emulsion were isotropic, which was the basis for the uniform grouping of the substructure units to create a multicavity structure, due to the high degree of uniformity within the particle sizes of the secondary emulsion. In contrast, no chambers were visible for the MCNs that were prepared without DEP emulsion (Figure S1a) in the comparative experiment. Similarly, for the primary emulsion without a high-speed shear force, the internal structure of the obtained MCNs was composed of a single cavity (Figure S1b), which verifies the fact that the use of oil phase (DEP) was the prerequisite for the formation of the internal chamber structure and the secondary emulsion was the basis for the establishment of the multi-chamber structure inside the NMMCNs.

**Figure 3:**
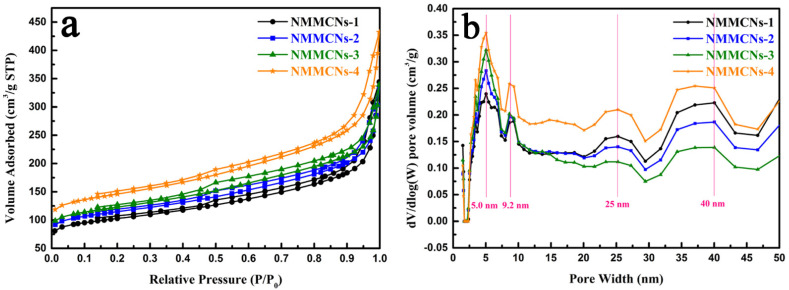
Nitrogen adsorption–desorption isotherms (**a**) and the corresponding PSD curves (**b**) of NMMCNs-1, NMMCNs-2, NMMCNs-3 and NMMCNs-4.

The pore characteristics of NMMCNs samples that were prepared with different contents of DEP were examined in N_2_ adsorption–desorption experiments. The N_2_ sorption isotherms, shown in Figure 3a, exhibited that all samples displayed typical type-IV isotherms with distinct hysteresis loops at the relative pressure of 0.4 < P/P_0_ < 0.8, which was consistent with the characteristics of mesoporous structures. Moreover, we could observe an H_3_ hysteresis loop at the relative pressure range of 0.85–1.0, confirming the existence of a hollow cavity inside the NMMCNs. Additionally, there were many types of mesopores (5.0, 9.2, 25 and 40 nm), as revealed by the PSD curves (Figure 3b). One type of mesopore that was centered at 5.0 nm stemmed from the removal of surfactant F127 during the high-temperature carbonization and the other type of mesopore, which was centered at 9.2 nm, was the result of the DEP swelling effect, which could be proven by the N_2_ adsorption isotherm and pore size distribution (PSD) curve with DEP = 0 (Figure S2). In the absence of DEP, only one peak that was centered at 5.0 nm could be detected. However, when a certain amount of DEP was added to the system, new mesopores with a size of 9.2 nm were formed, indicating that DEP had a swelling effect on F127 micelles. The swelling of F127 micelles resulted in an increase in the mesopore size. The analysis result of the PSD curve was relatively similar to the effect that was observed in the TEM image. Through the careful observation of the PSD curve, large mesopores at 25 nm and 40 nm could still be seen, which might have originated from the rough grooves on the surfaces of the particles. The BET-specific surface areas of NMMCNs-1, NMMCNs-2, NMMCNs-3 and NMMCNs-4 were calculated to be 334.1, 373.0, 399.9 and 474.1 m^2^ g^−1^ and the corresponding pore volumes were 0.45, 0.42, 0.43 and 0.55 cm^3^ g^−1^, respectively.

**Figure 4:**
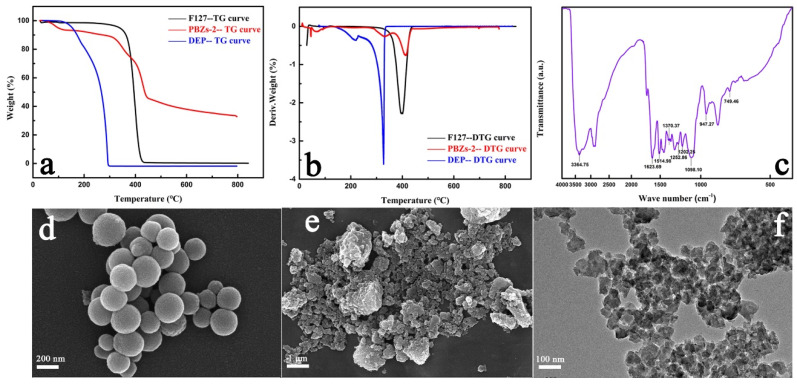
The TG curves (**a**) and corresponding DTG curves (**b**) of F127, DEP and PBZs-2, the FTIR spectrum (**c**) and SEM images (**d**) of colloidal PBZs-2 and the SEM (**e**) and TEM images (**f**) of MCNs without acid curing procedure.

Taking PBZs-2 as an example, TG analysis was employed to explore the pyrolysis behavior of PBZs-2 with cherimoya-shaped rough surfaces. The TG analysis (Figure 4a) curve of PBZs-2 comprises three stages of distinct weight loss. Obviously, the first stage was on the brink of 90–110 °C, typically due to the evaporation of water in the colloid. The second stage was at 220–310 °C, which might have been triggered by the pyrolysis of DEP according to the TG curve of DEP, and the third stage was between 380 and 410 °C, which was related to the pyrolysis of F127 when using the TG curve of F127 as a reference. To further dissect the position of the weightlessness peak in detail, calculus was performed on the three thermogravimetric curves to obtain the differential thermogravimetry (DTG) curves (Figure 4b). From the DTG curve of PBZs-2, the three weight loss processes corresponding to the TG curve could be easily obtained. From another perspective, both F127 and DEP participated triumphantly in the assembly process with carbon and nitrogen precursors. Furthermore, it could also be observed that the pyrolysis of F127 formed mesoporous aperture and that the successful pyrolysis of DEP was the key to the formation of the internal cavity structure. In order to verify that the obtained polymer nanoparticles had the structure of benzoxazine, the chemical structure of PBZs-2 was further confirmed by FTIR spectroscopy. As shown in Figure 4c, the absorption peaks at ~947 and 749 cm^−1^ were attributed to the vibration of the oxazine ring and the viscous vibration of the benzene ring, respectively [32,33]. The bands at 1252 cm^−1^and 1202 cm^−1^ could be linked to the asymmetric and symmetric stretching vibrations of the C–O–C group in the oxazine ring, respectively [34,35]. Additionally, the absorbance peak at ~1370 cm^−1^ was the stretching vibration of the C–N–C group on the oxazine ring, while the peaks at 1623 and 1514 cm^−1^ were the vibrations of the benzene ring skeleton vibration [32,35,36]. The strong peak band at ~3364 cm^−1^ might have been related to the stretching vibration of N–H [24,36]. The results described above adequately indicate the formation of benzoxazine. Additionally, the acid curing procedure was a crucial step in the overall synthesis process. In the previous reports and existing literature, the curing stage was completed by raising the temperature to stabilize the structure and morphology of the colloidal polymer [37,38]. In this research, the morphology of the particles was stabilized by the acid curing procedure to ensure that they remained in their original state during the subsequent high-temperature carbonization. To verify the effect of the acid curing procedure, MCNs were synthesized under the same conditions as the NMMCNs-2 without the acid curing program. As can be seen from the SEM and TEM images (Figure 4d–f), in the absence of the acid curing process, the morphology of the MCNs completely collapsed in the subsequent high-temperature carbonization process and there was no way to preserve its original shape. This can be explained by the fact that, under the initial synthesis conditions, the self-assembly of the polymeric precursor was only driven by hydrogen bond forces (the I^0^S^0^ mechanism) [39], the reaction temperature was relatively low and there was no way to achieve the temperature required for the polymer curing stage; thus, the structure of the formed PBZs resin was unstable in the subsequent carbonization process at 600 °C and the morphology could not be maintained. Nevertheless, the HCl acid curing procedure was introduced at the later stage of the reaction and through the participation of Cl^−^ as an intermediary, the structure of the PBZs resin was further strengthened and stabilized by the interaction of the Coulomb force (I^+^X^−^S^+^ mechanism) [4,14,40].

**Figure 5:**
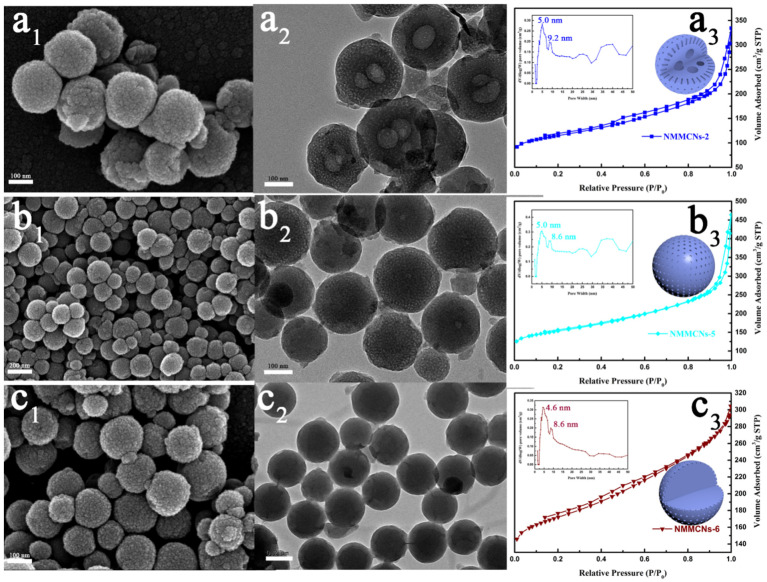
SEM images (**a_1_**–**c_1_**), TEM images (**a_2_**–**c_2_**), nitrogen adsorption–desorption isotherms (**a_3_**–**c_3_**) and corresponding PSD curves ((**a_3_**–**c_3_**), inset) of NMMCNs synthesized with different E/W ratios. (Row (**a**), NMMCNs-2; Row (**b**), NMMCNs-5; Row (**c**), NMMCNs-6).

A set of NMMCNs samples was synthesized by varying the quantitative relationship of ethanol and water (E/W) within the reaction system to explore the influence of the polarity of the solution on the morphology of the synthesized samples. As described in Figure 5, as the E/W ratio increased, the internal mesoscopic structures of the NMMCNs with cherimoya-shaped rough surfaces were modified significantly. When the E/W ratio rose from 5/35 to 10/30 and then to 15/25, the surfaces of the NMMCNs remained rough, while the internal cavities changed from multicavity to non-cavity structures (Figure 5a_2_–c_2_). This transformation can be explained by the fact that as the E/W ratio increased, the polarity of the solution decreased, which led to a higher solubility of DEP in water and offset the “emulsion swelling effect” that was produced by the DEP entering the PEO section of F127. Consequently, the contact area of the PBZs resin and DEP decreased, thereby making DEP unable to completely participate in the copolymerization of PBZs resin as an emulsion template. These results could be further verified by the N_2_ adsorption isotherm and the corresponding PSD curve (Figure 5a_3_–c_3_ and inset), in which the increase in the E/W ratio resulted in the decrease in the dual-mesopore size from 5.0 and 9.2 nm to 4.6 and 8.6 nm and also proved the reduction in the swelling effect of the emulsion. Hence, there were no chambers in the internal structure of the prepared NMMCNs-5 and NMMCNs-6. The morphology transformation could be attributed to the weakening of “the emulsion swelling effect”, which realized the internal mesoscopic structure transformation from multicavity to non-cavity mesoporous carbon nanospheres. Consequently, it can be concluded that an appropriate E/W ratio was an indispensable condition for the synthesis of particles with structural changes in the internal cavity.

**Figure 6:**
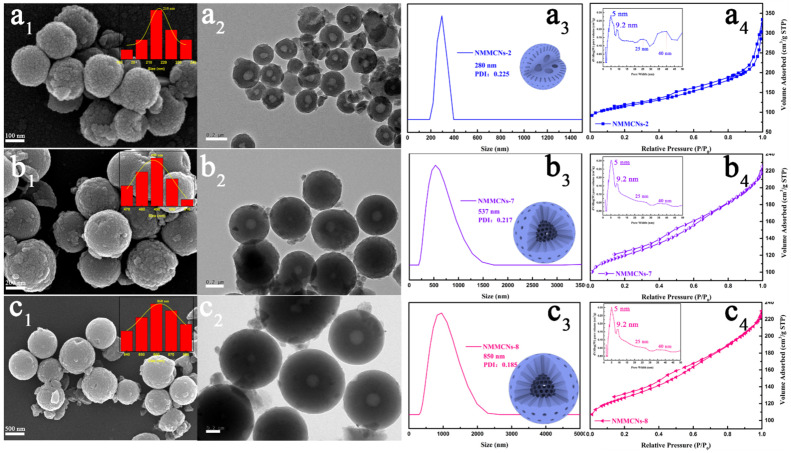
SEM images (**a1**–**c1**), TEM images (**a2**–**c2**), particle size distributions derived from DLS (**a3**–**c3**) and nitrogen adsorption–desorption isotherms (**a4**–**c4**), as well as the corresponding PSD curves ((**a4**–**c4**), inset) of NMMCNs synthesized with different EDA contents. (Row (**a**), NMMCNs-2; Row (**b**), NMMCNs-7; Row (**c**), NMMCNs-8).

Serving as a catalyst to synthesize benzoxazine resin and a precursor for N-doping, EDA played an irreplaceable role in this synthetic system. In order to demonstrate the effect of EDA, NMMCNs-7 and NMMCNs-8 were synthesized by increasing the content of EDA. Figure 6 exhibits the SEM and TEM images for those as synthesized by different doses of EDA with the other conditions being kept constant. Once the quantity of EDA was enhanced from 0.1 to 0.2 and then to 0.3 mL, the particle size of MCNs increased from 215 to 490 and then to 860 nm, accordingly. Astoundingly, the amount of EDA was positively correlated with the particle size. Through the linear fitting of EDA quantity and particle size (as shown in Figure S3), the equation y = 3225x − 123.33 with regression coefficient R^2^= 0.99 was obtained. Therefore, the diameter of the NMMCNs could be predicted by the equation, making it possible to programmatically alter the particle diameter by adjusting the amount of EDA. DLS (Figure 6a_3_–c_3_) revealed the same results. More surprisingly, through careful observation, the SEM images (Figure 6a_1_–c_1_) display that adjusting the amount of EDA could not only control the particle size, but also exert a certain influence on the surface roughness of the particles. A high dose of EDA made the DEP–F127–PBZ SSUs assemble within a large range instead of accumulating together in the form of small particles; thus, this resulted in the transformation of the NMMCNs surfaces from rough to smooth. The TEM images (Figure 6a_2_–c_2_) illustrate that the amount of EDA also had a definite impact on the internal cavities of the NMMCNs and the thickness of the shells. As the amount of EDA reached 0.2 mL, the multicavity internal structures of the NMMCNs completely disappeared, the internal cavities changed from multicavity to a single cavity (110 nm) with the shell thickness of 175 nm. By increasing the EDA to 0.3 mL, NMMCNs-8 particles with a single cavity size of 195 nm and a shell thickness of 330 nm were obtained, which also proved the role of EDA as a basic catalyst. EDA was the key factor in realizing the adjustment of the morphology from a smaller multicavity to a larger hollow sphere with an increasing diameter. The nitrogen adsorption–desorption isotherms of all of the prepared NMMCNs-2, NMMCNs-7 and NMMCNs-8 (Figure 6a_4_–c_4_) exhibited a type-IV curve with a definite capillary condensation in the P/P_0_ range of 0.4–0.8. The PSD curves (Figure 6a_4_–c_4_, insert) that were calculated using the Barrett–Joyner–Halenda (BJH) method revealed two mesopore sizes: 5.0 and 9.2 nm. It is noteworthy that pores of 25 nm and 40 nm were absent from the PSD curves for NMMCNs-7 and NMMCNs-8, which also proves that the rough grooves on the particle surfaces were gradually disappearing. This was consistent with the results of the SEM and TEM observations. With the increase in the EDA amount, the surfaces of NMMCNs-7 and NMMCNs-8 became slightly smoother. The S_BET_ values of NMMCNs-2, NMMCNs-7 and NMMCNs-8 were 374.0, 385.1 and 407.1 m^2^ g^−1^, respectively. The corresponding pore volumes were 0.42, 0.23 and 0.23 cm^3^ g^−1^, respectively.

**Figure 7:**
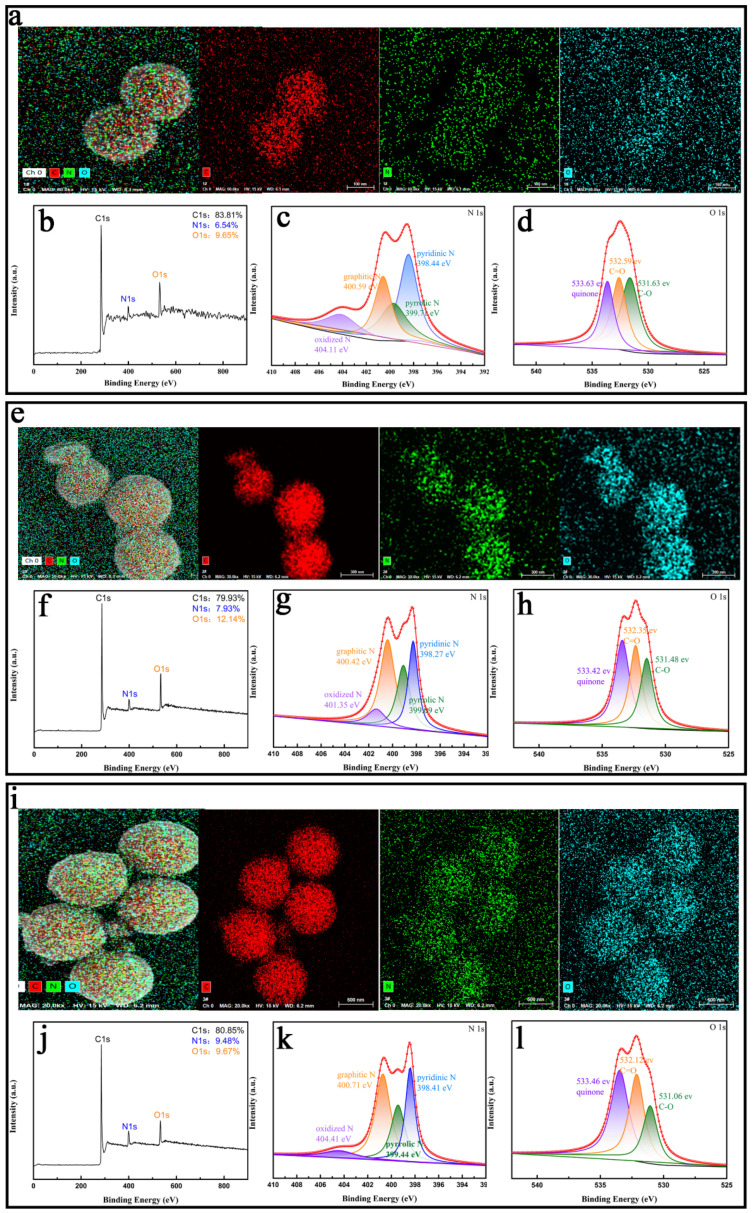
The (**a**) EDS element mapping analysis, (**b**) XPS survey spectrum, (**c**) high-resolution XPS spectra of N 1 s and (**d**) high-resolution XPS spectra of O 1 s for NMMCNs-2, the (**e**) EDS element mapping analysis, (**f**) XPS survey spectrum, (**g**) high-resolution XPS spectra of N 1 s and (**h**) high-resolution XPS spectra of O 1 s for NMMCNs-7 and the (**i**) EDS element mapping analysis, (**j**) XPS survey spectrum, (**k**) high-resolution XPS spectra of N 1 s and (**l**) high-resolution XPS spectra of O 1 s the NMMCNs-8.

To further assess the N-doping degree of MCNs when using EDA as a nitrogen source, an element mapping analysis and XPS analysis were used to explore the elementary distribution of samples with different EDA contents. Figure 7a,e,i presents the element mapping spectra of the NMMCNs samples with altered N-doped levels. The three samples were composed of C, N and O elements and they were distributed uniformly throughout the entire carbon skeleton matrix. The strongest signal of the three elements was carbon, suggesting that the prepared material was the typical carbonaceous material, but the signals of nitrogen and oxygen could also be clearly seen. Moreover, the XPS spectrum was employed to further investigate the element content of NMMCNs-2, NMMCNs-7 and NMMCNs-8. As depicted in Figure 7b, the XPS spectrum of the NMMCNs-2 showed three typical peaks of C 1 s, N 1 s and O 1 s without any impurities. The corresponding element contents were 83.81%, 6.54% and 9.65%, respectively, which were consistent with the results from the element mapping analysis. The corresponding high-resolution N 1 s XPS spectrum (Figure 7c) exhibited the presence of oxidized N (404.11 eV), graphitic N (400.59 eV), pyrrolic N (399.71 eV) and pyridinic N (398.44 eV) [26,41,42,43]. We also investigated the types of O atoms in the materials by fitting the O 1 s into three typical peaks at 533.63, 532.59 and 531.63 eV of the XPS spectrum (Figure 7d), which corresponded to quinone, C=O and C–O, respectively [17,34,42,43]. For NMMCNs-7 and NMMCNs-8, the surface N contents were measured as about 7.93% and 9.48% (Figure 7f,j), which also conformed to the measurements from the element mapping analysis. The fitting of the high-resolution N 1 s and O 1 s XPS spectrum (Figure 7g,h,k,l) also revealed the same four relative N species and three relative O species.

**Figure 8:**
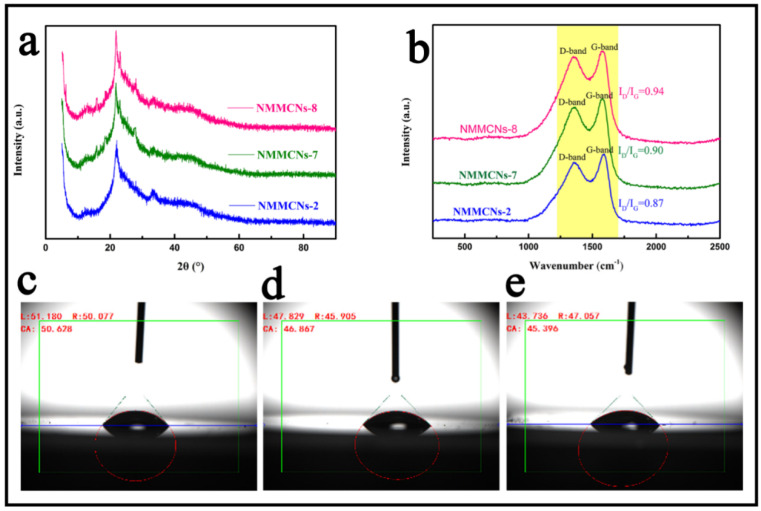
XRD patterns (**a**) and Raman spectra (**b**) of NMMCNs-2, NMMCNs-7 and NMMCNs-8 and (**c**), (**d**) and (**e**) correspond to the contact angles of NMMCNs-2, NMMCNs-7 and NMMCNs-8, respectively.

Additionally, the N-doping of the sample also affected the degree of the graphitization of the material. As shown in Figure 8a, the XRD patterns of NMMCNs-2, NMMCNs-7 and NMMCNs-8 showed two broad and low intensity diffraction peaks assigned to 002 (21.5°) and 100 (42°), indicating the disordered graphitization and amorphous nature of the carbonaceous structures [44,45]. To further confirm the continuous increase in N-doped content, Raman spectroscopy (Figure 8b) was carried out to investigate the degree of N-doping and graphitization. The D-band referred to the structural defects on the graphite plane in the carbonaceous material and the G-band was attributed to the E2g vibration mode in the sp^2^ bonds of the graphite crystallite [46,47]. The intensity ratio between the D-band and G-band (I_D_/I_G_) provided qualitative information about the degree of graphitization in the carbonaceous materials. The larger the I_D_/I_G_ ratio, the larger the structural defects in the carbonaceous material. As illustrated in Figure 8b, the I_D_/I_G_ values of NMMCNs-2, NMMCNs-7 and NMMCNs-8 were 0.87, 0.90 and 0.94, respectively. The increase in I_D_/I_G_ ratio indicates higher amounts of N-doped content and the introduction of a higher disorder level. It is worth noting that the N-doped and oxygen-containing functional groups could improve the surface hydrophilicity and wettability of the material, thus promoting its adsorption performance. Furthermore, the wettability of the as-prepared samples (NMMCNs-2, NMMCNs-7 and NMMCNs-8) were evaluated by water contact angle measurements using JYSP-180 equipment. The results are displayed in Figure 8c–e. Their average contact angles were 50.628, 46.867 and 45.396°, respectively, demonstrating that the increase in N-doped content could significantly improve the surface wettability and hydrophilicity of the material, thereby producing an excellent performance for the further application of the material.

### 3.2. Synthesis Mechanism

**Figure 9:**
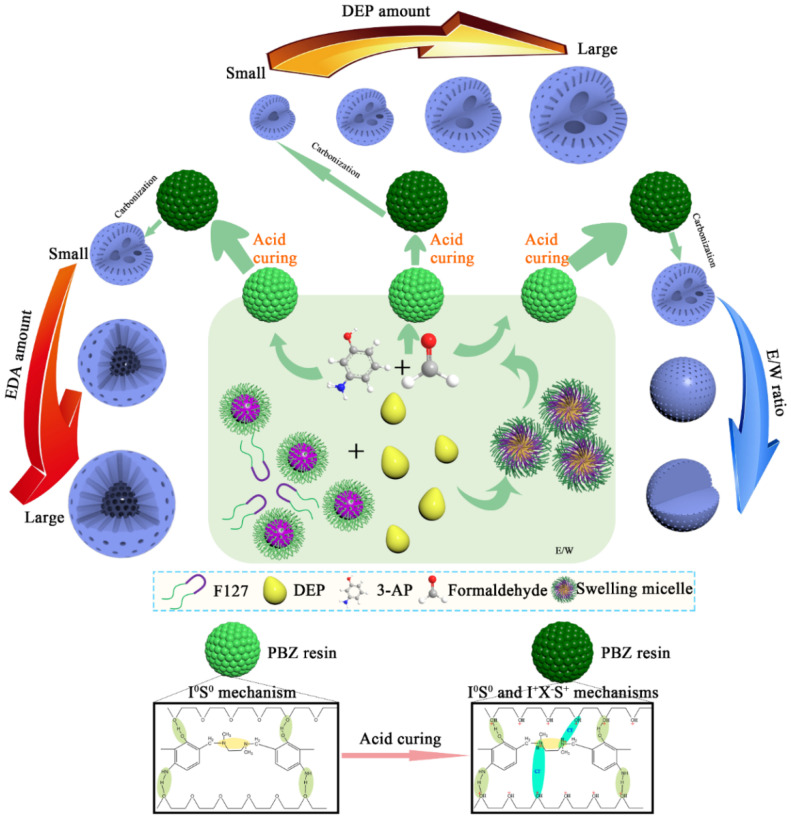
A schematic illustration for the formation mechanism of various NMMCNs-x.

Based on the above characterization results of NMMCNs with cherimoya-shaped rough surfaces and adjustable internal chambers, we propose an “emulsion swelling–acid curing mechanism” to explain its growth mechanism. As illustrated in Figure 9, the F127 triblock copolymer (PEO–PPO–PEO) was transformed into spherical micelles with the PPO segment as the core through a self-assembly process in an ethanol and water system. As the oil phase emulsion droplets, DEP penetrated the interior of the F127 micelles and formed the DEP–F127–H_2_O primary emulsion system under stirring. Significantly, the high-speed shearing force (16,000 rpm) of the homogenizer was employed to shear the primary emulsion into smaller emulsion droplets, thereby eventually constructing the secondary emulsion, in which the effect of Ostwald ripening on the merging of emulsion droplets was reduced by rapid temperature cooling [48]. Crucially, the high-speed shearing force caused the large droplets to stretch and eventually split into smaller droplets [49]. Consequently, the oil droplet size was minimized by the high-speed shearing force and the emulsion droplet size of the prepared secondary emulsion was extremely small and uniform. Since F127 is an amphiphilic triblock copolymer, DEP used as an oil phase could enter the core of the F127 hydrophobic segments and continuously swell, forming “swelling micelles” that could be used as templates to construct multi-chambers. Based on the secondary emulsion, the PEO segments of the PEO–PPO–PEO triblock copolymer interacted with the carbon and nitrogen precursors through the I^0^S^0^ mechanism under the action of hydrogen bond, then induce the gathering and pre-polymerization of PBZs resin on the surface of nanoemulsion droplets, thus forming DEP–F127–PBZs SSUs. Subsequently, the prepared DEP–F127–PBZ SSUs aggregated into the precursors of multi-chamber structures with different sizes through the action of surface-free energy. It is worth noting that the emulsion played a significant role as the prerequisite for the formation of the internal cavities and that the DEP–F127–PBZ SSUs were the basis for the configuration of the multicavity structures. Then, the acid curing process was carried out by pH adjustment using HCl. The addition of HCl reduced the pH value of the system and increased the hydrophobicity of the PPO segments, resulting in the swelling of the nanoemulsion. Meanwhile, the PEO segments also played a stabilizing role in this process [50]. The PBZs and PEO segments of F127 were protonated under acidic conditions, thereby inducing the three-component co-assembly that was used to promote the formation of F127^+^–Cl^−^–PBZ^+^. The participation of Cl^−^ as an intermediary could stabilize the structure of the DEP–F127–PBZ SSUs through the interaction of the Coulombic force (I^+^X^−^S^+^ mechanism), meaning that the hydrogen bond forces (I^0^S^0^ mechanism) and Coulombic forces (I^+^X^−^S^+^ mechanism) could coexist in the system and continue to strengthen the degree of cross-linking between the polymers, thus making the polymer even more stable. The subsequent process of high-temperature carbonization could still maintain its original shape. By increasing the amount of DEP, the interior structures of NMMCNs were modified from a single cavity to multicavity structures and the diameter of the cavities were gradually enlarged, which was the result of the further swelling of the DEP oil phase. Increasing the E/W ratio weakened the swelling effect of the emulsion droplets that were acting as the cavity template, so it was easy to realize the transformation from a multicavity to a non-cavity structure by controlling the E/W ratio. Moreover, the dosage of EDA also played a vital role in determining the polymerization and fusion rate of the DEP–F127–PBZ SSUs, further altering the mesostructures and particle sizes of the prepared NMMCNs. A large EDA content promoted the polymerization rate of the DEP–F127–PBZ SSUs, which in turn affected its stacking mode. In this situation, the DEP–F127–PBZ SSUs were assembled in an extensive range instead of stacking together in the form of small particles, thus forming larger and smoother NMMCNs.

### 3.3. Biological Effects

**Figure 10:**
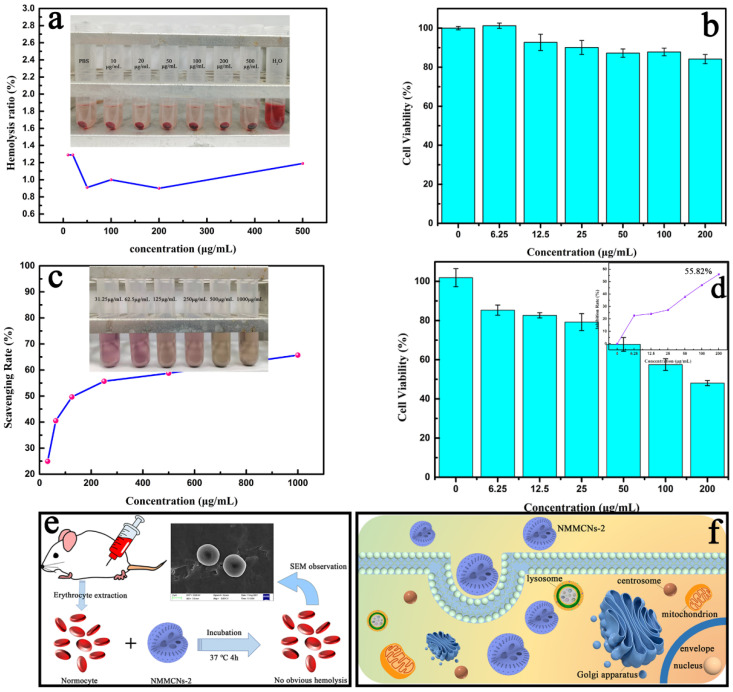
The hemolysis analysis (**a**) and cytotoxicity analysis (**b**) of NMMCNs-2, the in vitro antioxidation experiment analysis (**c**) and in vitro antitumor activity analysis (**d**) of Res–NMMCNs-2. The panel (**d**) inset is the analysis of the Hepa1-6 tumor cell inhibition rate of Res–NMMCNs-2 in vitro and (**e**) and (**f**) are schematic diagrams of the hemolysis experiment and cell uptake experiment, respectively.

**Figure 11:**
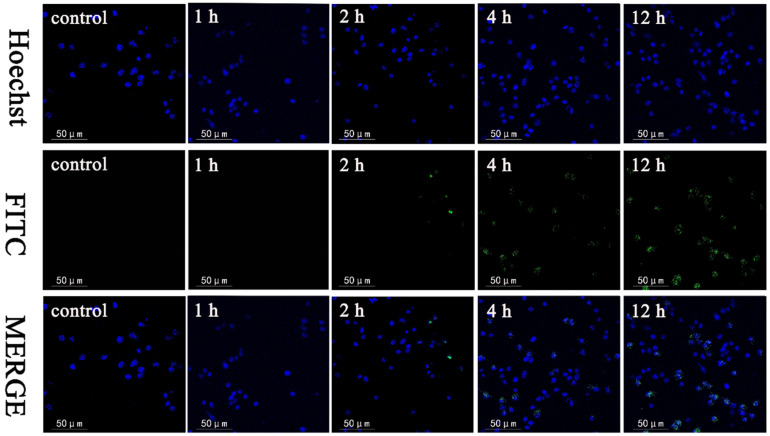
Confocal microscopy images of 3T3 cells (the control group) after treatment with FITC-grafted NMMCNs-2 for 1 h, 2 h, 4 h and 12 h.

The hemolysis test was performed to evaluate the blood compatibility of NMMCNs-2 as a drug carrier. According to the five concentration gradients of NMMCNs-2, the hemolysis rates with different concentration gradients were all lower than 2% after co-culturation with RBCs at 37 °C for 3 h (Figure 10a). It can also be observed from the SEM image (Figure 10e, insert) that the morphology of the RBCs was well maintained after co-incubation with NMMCNs-2, indicating that NMMCNs-2 exhibited glorious biocompatibility as a promising delivery platform in biological applications. Then, the cytotoxicity test of the prepared NMMCNs-2 with cherimoya-shaped rough surfaces was conducted by employing the cell lines of 3T3 cells as models for co-culturing 0–200 μg/mL of NMMCNs-2. The cytotoxicity assays (Figure 10b) illustrated that the cell viability exceeded 85% after 24 h, even at high concentrations (200 μg mL^−1^), demonstrating the low cytotoxicity of NMMCNs-2. In addition, cell uptake experiments were performed on 3T3 cells and FITC was grafted onto NMMCNs-2 as a fluorescent agent. The results of the fluorescence spectroscopy (Figure S4a) and UV-vis spectroscopy showed (Figure S4b) that FITC-grafted NMMCNs-2 were successfully prepared. It can be concluded from the confocal microscope image (Figure 11) that the degree of fluorescence within the cell was time-dependent. With time extension, the fluorescence intensity in the cell gradually increased. Therefore, it can be inferred that NMMCNs-2 could be well taken up by the cell to deliver drugs or biologically active molecules. Through the cytotoxicity and hemolytic tests, we understand that NMMCNs-2 could be beneficial in application as a drug carrier.

### 3.4. Drug Carrier

Resveratrol (Res) is a natural polyphenol compound that is known for its antioxidant, anti-inflammatory and anticancer effects [51]. However, the use of Res as a natural medicine with good application prospects is limited due to its instability, poor solubility and low bioavailability. In this research, the obtained NMMCNs-2 had an available mesoporous channel, small particle size with large mesopore and high specific surface area, which endowed it with extraordinary potential for drug delivery. NMMCNs-2 was utilized as a drug carrier to investigate the loading capacity of NMMCNs-2 and its improved performance regarding the dissolution and release rate of Res. The drug loading of the Res–NMMCNs-2 delivery system with distinct drug loading ratios is listed in Figure S6a. The XRD patterns (Figure S6b) revealed that the typical crystal peak of Res gradually disappeared with the increase in the drug loading ratio, proving that Res was moderately loaded into NMMCNs-2. When the drug loading ratio reached 1:2, all of the crystal peaks of Res disappeared, indicating that all of the Res was loaded into the mesoporous materials. At the same time, it could also explain why the drug loading became smaller and smaller as the drug loading ratio increased. The same experimental results were apparent in the FTIR spectrum (Figure S6c). Additionally, the influence of methanol as a solvent should be taken into account. From the SEM images, Res and the recrystallized Res both showed a crystalline state (Figure S7a,b), indicating that the methanol solution had no effect on the crystal shape of Res, which was consistent with the XRD pattern. Combined with the XRD pattern and FTIR spectrum analysis, the optimal drug loading ratio of Res and NMMCNs-2 was determined to be 1:2. On the other hand, in the DSC spectrum (Figure S6d), a sharp endothermic peak was observed at 271 °C, attesting to the crystal state of the pure Res. However, no endothermic peak emerged on the DSC curve of Res–NMMCNs-2. Depending on the above characterization, Res completely transformed from a crystalline to amorphous state when Res was loaded into NMMCNs-2, thus improving the solubility and bioavailability of Res.

The DPPH• scavenging capacity of the Res–NMMCNs-2 delivery system was measured (Figure 10c). The results illustrated that the Res–NMMCNs-2 delivery system still maintained the antioxidant activity of Res. The DPPH• scavenging capacity gradually increased with the increasing concentration of Res–NMMCNs-2 and reached as high as 77.32% at 1 mg/mL. In the inset illustration of Figure 10c, we can observe a color change from purplish to yellow, which also confirms the gradual increase in DPPH• scavenging capacity. NMMCNs-2 with the same concentrations dispersed in an ethanol solution were employed as the comparative samples to determine their antioxidant capacity. As demonstrated in Figure S8, the material itself did not demonstrate any antioxidant activity, indicating that the antioxidant capacity of the Res–NMMCNs-2 delivery system was the result of Res release. The in vitro antitumor activity study of Res–NMMCNs-2 also revealed similar results (Figure 10d). When the Res–NMMCNs-2 delivery system at a 200 μg/mL concentration was co-cultured with mouse liver cancer cells (Hepa1-6) for 24 h, the tumor cell inhibition rate reached 55.82% (Figure 10d, inset). The successful implementation of the Res–NMMCNs-2 delivery system could open the door to a brand new world for the application of mesoporous materials in the field of biomedicine.

**Figure 12:**
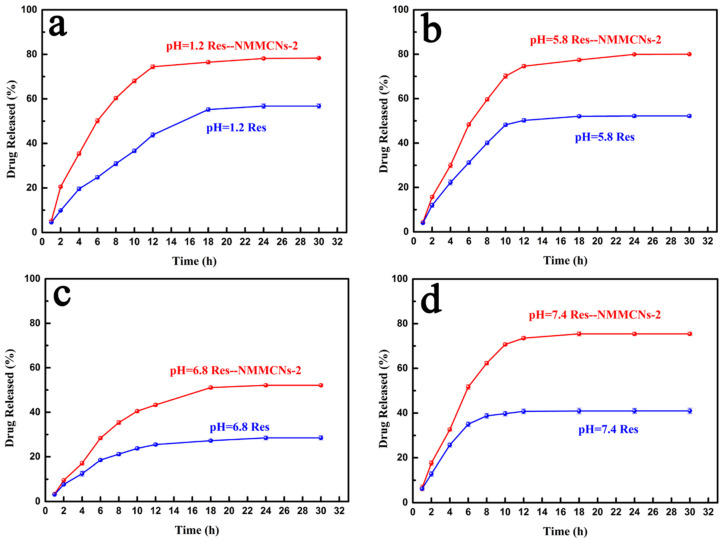
The drug release curves of pure Res and Res–NMMCNs-2 at different pH values ((**a**), pH = 1.2; (**b**), pH = 5.8; (**c**), pH = 6.8; (**d**), pH = 7.4).

In order to investigate the dissolution and release of pure drugs through Res and the Res–NMMCNs-2 delivery system, in vitro drug release experiments were conducted in buffer solutions of pH 1.2, 5.8, 6.8 and 7.4 to simulate human gastric juice, tumor cell internal environments, intestinal juice and body fluids, respectively. The release profiles (Figure 12) manifested that the Res–NMMCNs-2 delivery system could significantly improve the pure Res release. This result was attributed to the spatial restriction effect of the mesoporous channel of NMMCNs-2, which prevented the loaded Res from recrystallizing and provoked it to transform into an amorphous state, thereby improving the dissolution and release properties of the pure Res. The above observation indicates that the NMMCNs-2 delivery system could load poorly soluble drugs and improve their bioavailability. Five mathematical models were employed to fit the release experimental data of Res and the Res–NMMCNs-2 delivery systems in buffer solutions of different pH values. The diagrams presenting the fitting of the Res and Res–NMMCNs-2 release data with kinetic models are included in the Supporting Information (Figures S9–S12). The calculated kinetic values of the correlation coefficient (R^2^) and equations are presented in Table S2. The correlation coefficients of the Weibull equation obtained by Res–NMMCNs-2 in the pH 1.2, 5.8, 6.8 and 7.4 buffer media were 0.9919, 0.9970, 0.9970 and 0.9978, respectively. By comparing the in vitro release curves of the pure drug Res and Res–NMMCNs-2, they were more in line with the Weibull model, indicating that the release of Res and Res–NMMCNs-2 needs to go through two stages: the rapid release phase and the slow release phase.

## 4. Conclusions

In summary, we presented a novel synthesis strategy based on the “emulsion swelling–acid curing mechanism” to prepare NMMCNs. By using 3-AP, formaldehyde and EDA as the precursors of PBZs resin, a series of NMMCNs with cherimoya-shaped rough surfaces and adjustable cavities was synthesized by the polymerization of the PBZs resin with a secondary emulsion prepared under high-speed shearing force conditions. Impressively, the directional transformation of the internal structures and particle sizes of the NMMCNs could be accomplished by adjusting the synthesis parameters. For example, by adjusting the content of DEP, the multicavity size of NMMCNs could be tuned; varying the E/W ratio could accomplish the transformation of the internal multicavity structures to non-cavity; employing different doses of EDA achieved the conversion of multicavity to single cavity structures. Interestingly, the EDA dosage gradient experiment realized the prediction of the linear relationship between particle size and EDA amount, making it possible to programmatically alter the particle diameter. Furthermore, the traditional heat curing step was abandoned and a novel acid curing procedure was adopted to achieve the perfect morphology preservation of polymer colloidal particles during the subsequent high-temperature carbonization process. As a nano-delivery system, NMMCNs-2 exhibited low cytotoxicity, no hemolysis and could be completely taken up by 3T3 cells, indicating an outstanding biocompatibility. When loaded with the drug Res, the Res–NMMCNs-2 delivery system could exert the antitumor activity and antioxidant activity of pure Res. Moreover, the Res–NMMCNs-2 delivery system exhibited pH responsibility and could improve the release behavior of pure Res. Based on the calculations of five drug release models, the drug release rule of the NMMCNS-2 delivery system and pure Res under different pH conditions were most consistent with the Weibull model, including the release mechanism comprising a fast release stage and a slow release stage.

## Figures and Tables

**Table 1:** The synthesis parameters of PBZs-x and NMMCNs-x.

Samples		Synthesis Parameters			
Before Carbonization	After Carbonization	F127 (g)	DEP (mL)	Ethanol/Water E/W (*v*/*v*)	EDA (mL)
PBZs-1	NMMCNs-1	0.4	0.1	5/35	0.1
PBZs-2	NMMCNs-2	0.4	0.2	5/35	0.1
PBZs-3	NMMCNs-3	0.4	0.3	5/35	0.1
PBZs-4	NMMCNs-4	0.4	0.4	5/35	0.1
PBZs-5	NMMCNs-5	0.4	0.3	10/30	0.1
PBZs-6	NMMCNs-6	0.4	0.3	15/25	0.1
PBZs-7	NMMCNs-7	0.4	0.3	5/35	0.2
PBZs-8	NMMCNs-8	0.4	0.3	5/35	0.3

## Data Availability

All data reported in this paper are contained within the manuscript.

## Supplementary Materials

The following supporting information can be downloaded at: https://www.mdpi.com/article/10.3390/ma15072591/s1, Table S1: Kinetic models applied to describe the release of Res from mesoporous materials, Figure S1: TEM image (a) of solid sphere MCNs prepared in the absence of DEP and TEM image (b) of single cavity MCNs prepared without high-speed shearing force, Figure S2: Nitrogen adsorption–desorption isotherm of (a) MCNs prepared in the absence of DEP and (b) the corresponding PSD curve, Figure S3: The linear relationship between the amount of EDA and the particle size of NMMCNs, Figure S4: The fluorescence spectrum (a) and UV-vis spectrum (b) of FITC, NMMCNs-2 and FITC-grafted NMMCNs-2, Figure S5: The drug loading charts (a), XRD patterns (b) and FTIR spectrums (c) of Res–NMMCNs-2 with different drug loading ratios and DSC curves (d) of the pure Res, NMMCNs-2 and Res–NMMCNs-2, Figure S6: SEM images of Res and recrystallized Res in methanol, Figure S7: Antioxidant activity of pure material NMMCNs-2, Figure S8: Fitting of release data performed at pH 1.2 for Res and Res–NMMCNs-2 to zero order (a), first order (b), Higuchi (c), Weibull (d) and Ritger–Peppas (e) models, Figure S9: Fitting of release data performed at pH 5.8 for Res and Res–NMMCNs-2 to zero order (a), first order (b), Higuchi (c), Weibull (d) and Ritger–Peppas (e) models, Figure S10: Fitting of release data performed at pH 6.8 for Res and Res–NMMCNs-2 to zero order (a), first order (b), Higuchi (c), Weibull (d) and Ritger–Peppas (e) models, Figure S11: Fitting of release data performed at pH 7.4 for Res and Res–NMMCNs-2 to zero order (a), first order (b), Higuchi (c), Weibull (d) and Ritger–Peppas (e) models.

## References

[1] Yu R., Huang X., Liu Y., Kong Y., Gu Z., Yang Y., Wang Y., Ban W., Song H., Yu C. (2020). Shaping Nanoparticles for Interface Catalysis: Concave Hollow Spheres via Deflation-Inflation Asymmetric Growth. Adv. Sci..

[2] Tao Q., Zhu Z., Ye S., Lin G., Chen H., Tu Y., Bai G., Zhang L., Yang X. (2021). Surface Polymerization and Controlled Pyrolysis: Tailorable Synthesis of Bumpy Hollow Carbon Spheres for Energy Storage. Langmuir.

[3] Zhu X., Xia Y., Zhang X., Al-Khalaf A.A., Zhao T., Xu J., Peng L., Hozzein W.N., Li W., Zhao D. (2019). Synthesis of carbon nanotubes@mesoporous carbon core–shell structured electrocatalysts via a molecule-mediated interfacial co-assembly strategy. J. Mater. Chem. A.

[4] Xie H., Zhao Y., Tian Y., Wang X., Yan M. (2019). Tailored synthesis from rhombic dodecahedron to spherical ordered mesoporous carbon nanoparticles via one-step strategy. Carbon.

[5] Tian Z., Jiang H., Huang M., Wang G.H. (2020). Facile Synthesis of Size-Controlled Nitrogen-Doped Mesoporous Carbon Nanosphere Supported Ultrafine Ru Nanoparticles for Selective Hydrogenation of Quinolines. Chemistry.

[6] Fei H.F., Long Y., Yu H.J., Yavitt B.M., Fan W., Ribbe A., Watkins J.J. (2020). Bimodal Mesoporous Carbon Spheres with Small and Ultra-Large Pores Fabricated Using Amphiphilic Brush Block Copolymer Micelle Templates. ACS Appl. Mater. Interfaces.

[7] Huang W., Xie H., Tian Y., Wang X. (2018). Controlled Growth of N-Doped and Large Mesoporous Carbon Spheres with Adjustable Litchi-Like Surface and Particle Size as a Giant Guest Molecule Carrier. ACS Appl. Mater. Interfaces.

[8] Liu J., Xie L., Deng J., Gong Y., Tang G., Bai H., Wang Y. (2019). Annular Mesoporous Carbonaceous Nanospheres from Biomass-Derived Building Units with Enhanced Biological Interactions. Chem. Mater..

[9] Peng J., Zhang W., Yu P., Pang H., Liang Y. (2020). Improved ion-diffusion performance by engineering an ordered mesoporous shell in hollow carbon nanospheres. Chem. Comm..

[10] Lu A.H., Sun T., Li W.C., Sun Q., Han F., Liu D.H., Guo Y. (2011). Synthesis of discrete and dispersible hollow carbon nanospheres with high uniformity by using confined nanospace pyrolysis. Angew. Chem. Int. Engl..

[11] Song Y., Li Z., Guo K., Shao T. (2016). Hierarchically ordered mesoporous carbon/graphene composites as supercapacitor electrode materials. Nanoscale.

[12] Wang M., Wang X., Yue Q., Zhang Y., Wang C., Chen J., Cai H., Lu H., Elzatahry A.A., Zhao D. (2014). Templated Fabrication of Core–Shell Magnetic Mesoporous Carbon Microspheres in 3-Dimensional Ordered Macroporous Silicas. Chem. Mater..

[13] Shen G., Sun X., Zhang H., Liu Y., Zhang J., Meka A., Zhou L., Yu C. (2015). Nitrogen-doped ordered mesoporous carbon single crystals: Aqueous organic–organic self-assembly and superior supercapacitor performance. J. Mater. Chem. A.

[14] Zhu X., Wang S., Huang W., Tian Y., Wang X. (2016). Controllable synthesis of mesoporous carbon nanospheres with uniform size by a facile one-pot aqueous strategy under highly acidic conditions. Carbon.

[15] Shao W., Hu F., Liu S., Zhang T., Song C., Weng Z., Wang J., Jian X. (2021). Carbon spheres with rational designed surface and secondary particle-piled structures for fast and stable sodium storage. J. Energy Chem..

[16] Zhang F., Zong S., Zhang Y., Lv H., Liu X., Du J., Chen A. (2021). Preparation of hollow mesoporous carbon spheres by pyrolysis-deposition using surfactant as carbon precursor. J. Power Sources.

[17] Liu J., Zhao Y., Xu L., Wang X., Tian Y. (2020). Dual soft-templated congenerous assembly to raspberry-shaped mesoporous carbon nanoparticles with hierarchical mesopores. Microporous Mesoporous Mater..

[18] Zhang L.-H., He B., Li W.-C., Lu A.-H. (2017). Surface Free Energy-Induced Assembly to the Synthesis of Grid-Like Multicavity Carbon Spheres with High Level In-Cavity Encapsulation for Lithium-Sulfur Cathode. Adv. Energy Mater..

[19] Chen C., Wang H., Han C., Deng J., Wang J., Li M., Tang M., Jin H., Wang Y. (2017). Asymmetric Flasklike Hollow Carbonaceous Nanoparticles Fabricated by the Synergistic Interaction between Soft Template and Biomass. J. Am. Chem. Soc..

[20] Yang X., Lu P., Yu L., Pan P., Elzatahry A.A., Alghamdi A., Luo W., Cheng X., Deng Y. (2020). An Efficient Emulsion-Induced Interface Assembly Approach for Rational Synthesis of Mesoporous Carbon Spheres with Versatile Architectures. Adv. Funct. Mater..

[21] Wang T., Sun Y., Zhang L., Li K., Yi Y., Song S., Li M., Qiao Z.A., Dai S. (2019). Space-Confined Polymerization: Controlled Fabrication of Nitrogen-Doped Polymer and Carbon Microspheres with Refined Hierarchical Architectures. Adv. Mater..

[22] Yu X.-F., Li W.-C., Hu Y.-R., Ye C.-Y., Lu A.-H. (2021). Sculpturing solid polymer spheres into internal gridded hollow carbon spheres under controlled pyrolysis micro-environment. Nano Res..

[23] Zhang Z., Jia B., Liu L., Zhao Y., Wu H., Qin M., Han K., Wang W.A., Xi K., Zhang L. (2019). Hollow Multihole Carbon Bowls: A Stress-Release Structure Design for High-Stability and High-Volumetric-Capacity Potassium-Ion Batteries. ACS Nano.

[24] Wang S., Li W.C., Hao G.P., Hao Y., Sun Q., Zhang X.Q., Lu A.H. (2011). Temperature-programmed precise control over the sizes of carbon nanospheres based on benzoxazine chemistry. J. Am. Chem. Soc..

[25] Hao G.-P., Li W.-C., Qian D., Wang G.-H., Zhang W.-P., Zhang T., Wang A.-Q., Schüth F., Bongard H.-J., Lu A.-H. (2011). Structurally Designed Synthesis of Mechanically Stable Poly(benzoxazine-co-resol)-Based Porous Carbon Monoliths and Their Application as High-Performance CO2Capture Sorbents. J. Am. Chem. Soc..

[26] Konnola R., Anirudhan T.S. (2020). Efficient carbon dioxide capture by nitrogen and sulfur dual-doped mesoporous carbon spheres from polybenzoxazines synthesized by a simple strategy. J. Environ. Chem. Eng..

[27] Manmuanpom N., Thubsuang U., Dubas S.T., Wongkasemjit S., Chaisuwan T. (2018). Enhanced CO_2_ capturing over ultra-microporous carbon with nitrogen-active species prepared using one-step carbonization of polybenzoxazine for a sustainable environment. J. Environ. Manag..

[28] Li Y., Qi J., Li J., Shen J., Liu Y., Sun X., Shen J., Han W., Wang L. (2017). Nitrogen-Doped Hollow Mesoporous Carbon Spheres for Efficient Water Desalination by Capacitive Deionization. ACS Sustain. Chem. Eng..

[29] Zhao Y., Lyu H., Liu Y., Liu W., Tian Y., Wang X. (2021). Rational design and synthesis of multimorphology mesoporous carbon@silica nanoparticles with tailored structure. Carbon.

[30] Yao Y., Ye H., Qi Z., Mo L., Yue Q., Baral A., Hoon D.S.B., Vera J.C., Heiss J.D., Chen C.C. (2016). B7-H4(B7x)-Mediated Cross-talk between Glioma-Initiating Cells and Macrophages via the IL6/JAK/STAT3 Pathway Lead to Poor Prognosis in Glioma Patients. Clin. Cancer Res. Off. J. Am. Assoc. Cancer Res..

[31] Wu J., Wang Y., Yang H., Liu X., Lu Z. (2017). Preparation and biological activity studies of resveratrol loaded ionically cross-linked chitosan-TPP nanoparticles. Carbohydr. Polym..

[32] Zhang H., Xu L., Liu G. (2020). Synthesis of Benzoxazine-Based N-Doped Mesoporous Carbons as High-Performance Electrode Materials. Appl. Sci..

[33] Zhao J., Gilani M.R.H.S., Lai J., Nsabimana A., Liu Z., Luque R., Xu G. (2018). Autocatalysis Synthesis of Poly(benzoxazine-co-resol)-Based Polymer and Carbon Spheres. Macromolecules.

[34] Wan L., Wang J., Xie L., Sun Y., Li K. (2014). Nitrogen-enriched hierarchically porous carbons prepared from polybenzoxazine for high-performance supercapacitors. ACS Appl. Mater. Interfaces.

[35] Gao X., Sun X., Xu L., Zhang H., Liu G. (2019). Synthesis and electrochemical properties of benzoxazine-based heteroatom-doped carbon materials. J. Mater..

[36] Wan L., Wang J., Feng C., Sun Y., Li K. (2015). Synthesis of polybenzoxazine based nitrogen-rich porous carbons for carbon dioxide capture. Nanoscale.

[37] Liu D., Zhang L., Lv P. (2020). Facile synthesis of ordered mesoporous carbon monolith with close-packed microspheres structure through emulsion templating. Microporous Mesoporous.

[38] Yue F., Gao G., Li F., Zheng Y., Hou S. (2018). Size-controlled synthesis of urchin-like reduced graphene oxide microspheres with highly packed density by emulsion-assisted in-situ assembly and their supercapacitor performance. Carbon.

[39] Hu T., Li Y., Gao W., Wang X., Tian Y. (2019). Engineering of rich nitrogen-doped and magnetic mesoporous carbon nanospheres with predictable size uniformity for acid dye molecules adsorption. Microporous Mesoporous.

[40] Chong J., Zhu X., Huang W., Wang X., Tian Y. (2018). The fabrication of size-tunable nitrogen-doped dual-mesoporous carbon nanospheres with excellent thermal stability via colloidal silica driving co-assembly strategy. Carbon.

[41] Liu J., Yang T., Wang D.W., Lu G.Q., Zhao D., Qiao S.Z. (2013). A facile soft-template synthesis of mesoporous polymeric and carbonaceous nanospheres. Nat. Commun..

[42] Du J., Chen A., Liu L., Li B., Zhang Y. (2020). N-doped hollow mesoporous carbon spheres prepared by polybenzoxazines precursor for energy storage. Carbon.

[43] Zhao R., Jin Z., Wang J., Zhang G., Zhang D., Sun Y., Guan T., Zhao J., Li K. (2018). Adsorptive desulfurization of model fuel by S, N-codoped porous carbons based on polybenzoxazine. Fuel.

[44] Li W., Li B., Shen M., Gao Q., Hou J. (2020). Use of Gemini surfactant as emulsion interface microreactor for the synthesis of nitrogen-doped hollow carbon spheres for high-performance supercapacitors. Chem. Eng. J..

[45] Liu Y., Wang Z., Teng W., Zhu H., Wang J., Elzatahry A.A., Al-Dahyan D., Li W., Deng Y., Zhao D. (2018). A template-catalyzed in situ polymerization and co-assembly strategy for rich nitrogen-doped mesoporous carbon. J. Mater. Chem. A.

[46] Peng L., Hung C.T., Wang S., Zhang X., Zhu X., Zhao Z., Wang C., Tang Y., Li W., Zhao D. (2019). Versatile Nanoemulsion Assembly Approach to Synthesize Functional Mesoporous Carbon Nanospheres with Tunable Pore Sizes and Architectures. J Am. Chem. Soc..

[47] Zhou T., Yao Z., Ma R., Zhou Z., Liu G., Qian L., Zhu Y., Wang J. (2016). In situ formation of nitrogen-doped carbon nanoparticles on hollow carbon spheres as efficient oxygen reduction electrocatalysts. Nanoscale.

[48] Taylor P. (2003). Ostwald ripening in emulsions: Estimation of solution thermodynamics of the disperse phase. Adv. Colloid Interface Sci..

[49] Long D., Lu F., Zhang R., Qiao W., Zhan L., Liang X., Ling L. (2008). Suspension assisted synthesis of triblock copolymer-templated ordered mesoporous carbon spheres with controlled particle size. Chem. Comm..

[50] Liu X., Song P., Hou J., Wang B., Xu F., Zhang X. (2018). Revealing the Dynamic Formation Process and Mechanism of Hollow Carbon Spheres: From Bowl to Sphere. ACS Sustain. Chem. Eng..

[51] Carter L.G., D’Orazio J.A., Pearson K.J. (2014). Resveratrol and cancer: Focus on in vivo evidence. Endocr.-Relat. Cancer.

