# Supplementary material for: Nanoengineering of N-doped Mesoporous Carbon Nanoparticles with Adjustable Internal Cavities via Emulsion-Induced Assembly
Source: Materials (Basel). 2022 Apr 1;15(7):2591. doi: 10.3390/ma15072591 (PMC9000577; doi:10.3390/ma15072591)
Supplement: Supplementary file 1 [file materials-15-02591-s001.zip › materials-1620900-supplementary.pdf]

materials

MDPI

Supplementary Materials

Nanoengineering of N-doped Mesoporous Carbon Nanoparticles with Adjustable Internal Cavities via Emulsion-induced Assembly

Cong Wang [1], Xiaoxi Zhao [1] Xiufang Wang [1,*] and Yong Tian [1,*]

[1] Guangdong Provincial Engineering Center of Topical Precise Drug Delivery System, School of Pharmacy, Guangdong Pharmaceutical University, Guangzhou, China, 510006; wangcong199662@163.com (C.W.); zhaoxx_17@163.com (X.Z.)

* Correspondence: x_f_wang@163.com (X.W.); tian_yong_tian@163.com

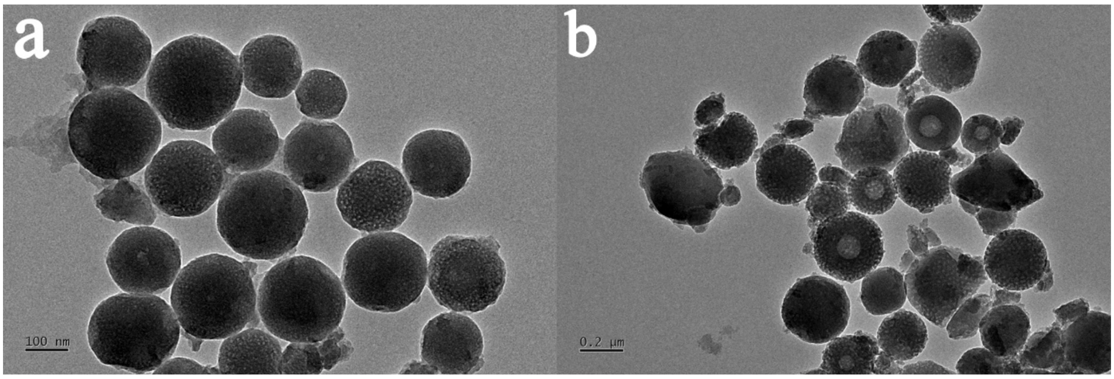

Figure S1. TEM image (a) of solid sphere MCNs prepared in the absence of DEP and TEM image (b) of single cavity MCNs prepared without high-speed shearing force.

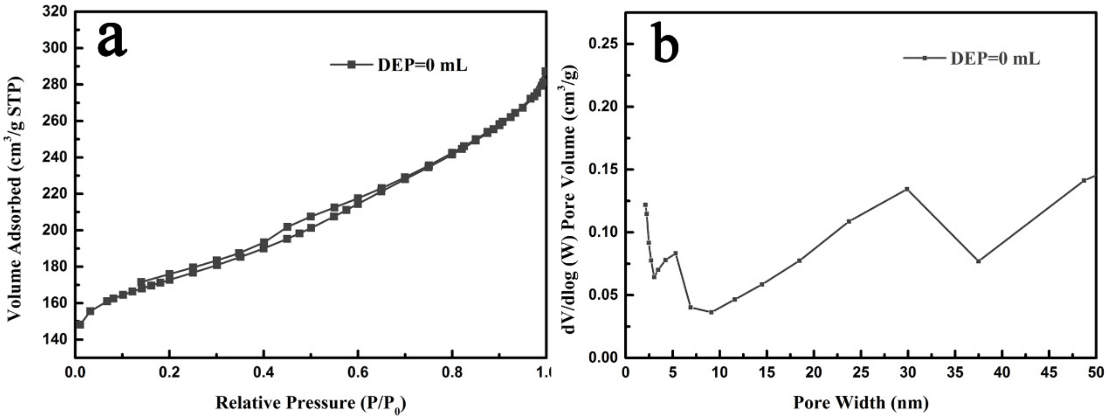

Figure S2. Nitrogen adsorption–desorption isotherm of (a) MCNs prepared in the absence of DEP and (b) the corresponding PSD curve.

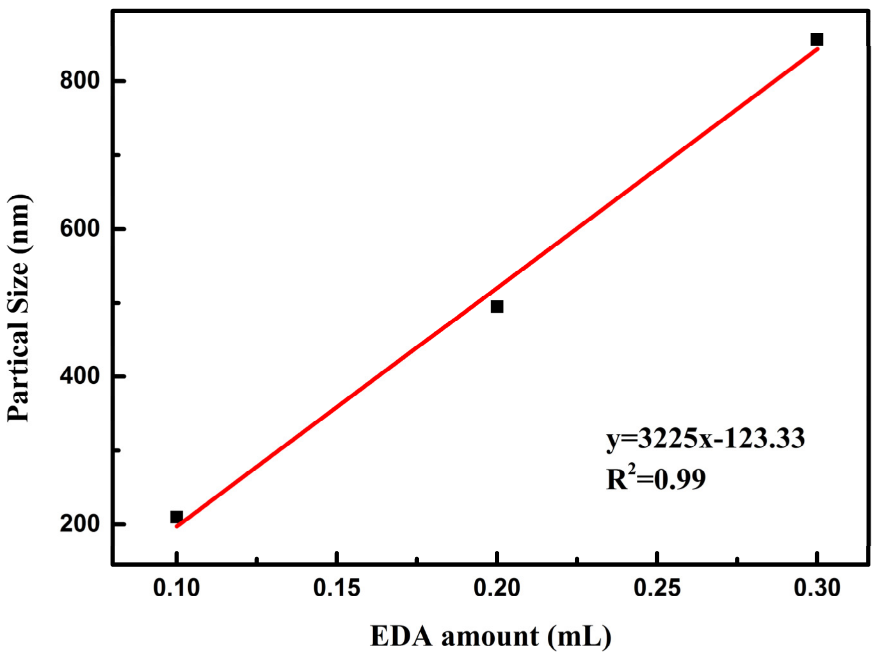

Figure S3. The linear relationship between the amount of EDA and the particle size of NMMCNs

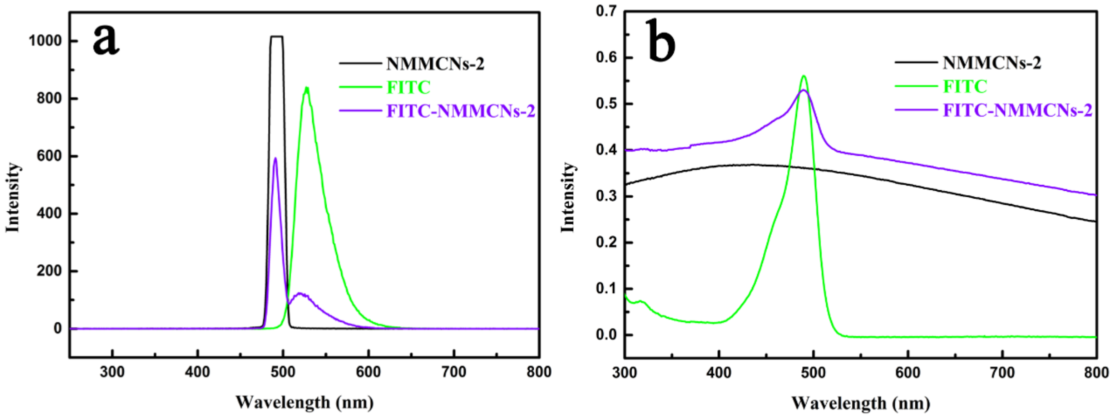

Figure S4. The fluorescence spectrum (a) and UV-vis spectrum (b) of FITC, NMMCNs-2 and FITC-grafted NMMCNs-2.

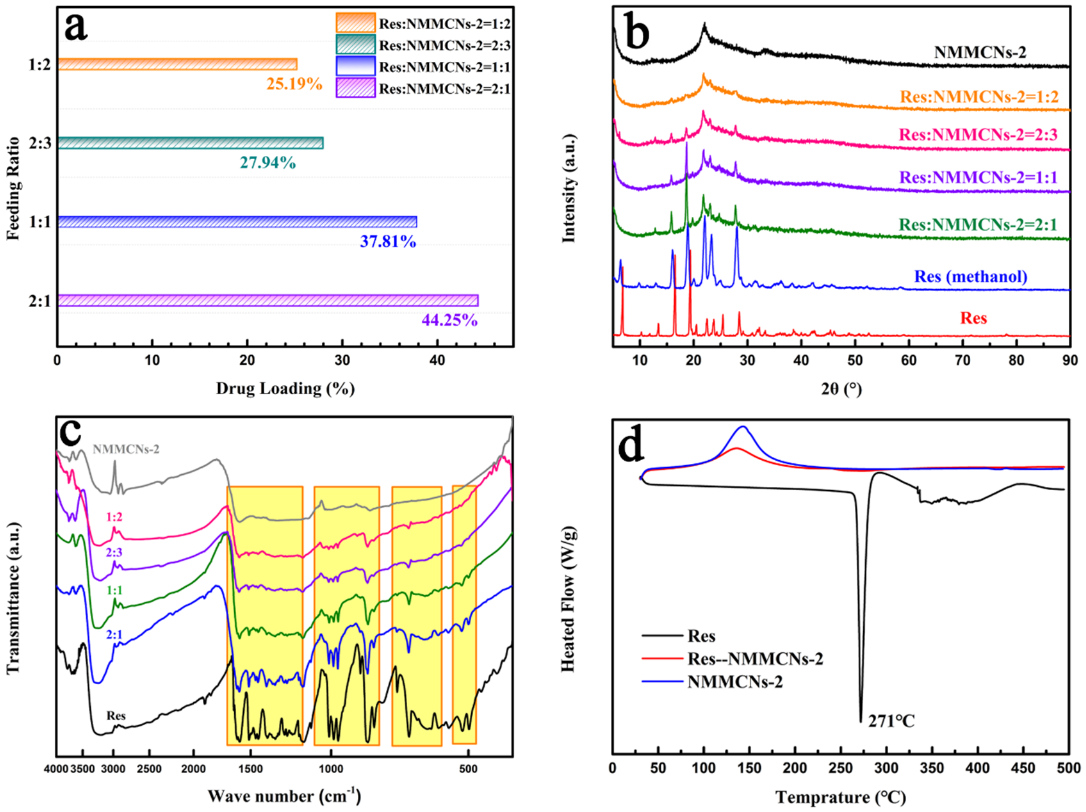

Figure S5. The drug loading charts (a), XRD patterns (b) and FTIR spectrums (c) of Res–NMMCNs-2 with different drug loading ratios and DSC curves (d) of the pure Res, NMMCNs-2 and Res–NMMCNs-2.

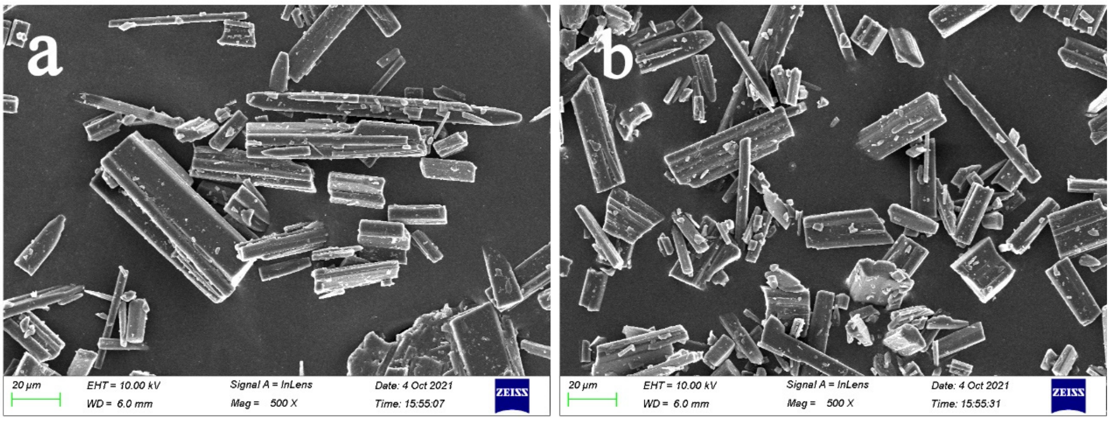

Figure S6. SEM images of Res and recrystallized Res in methanol.

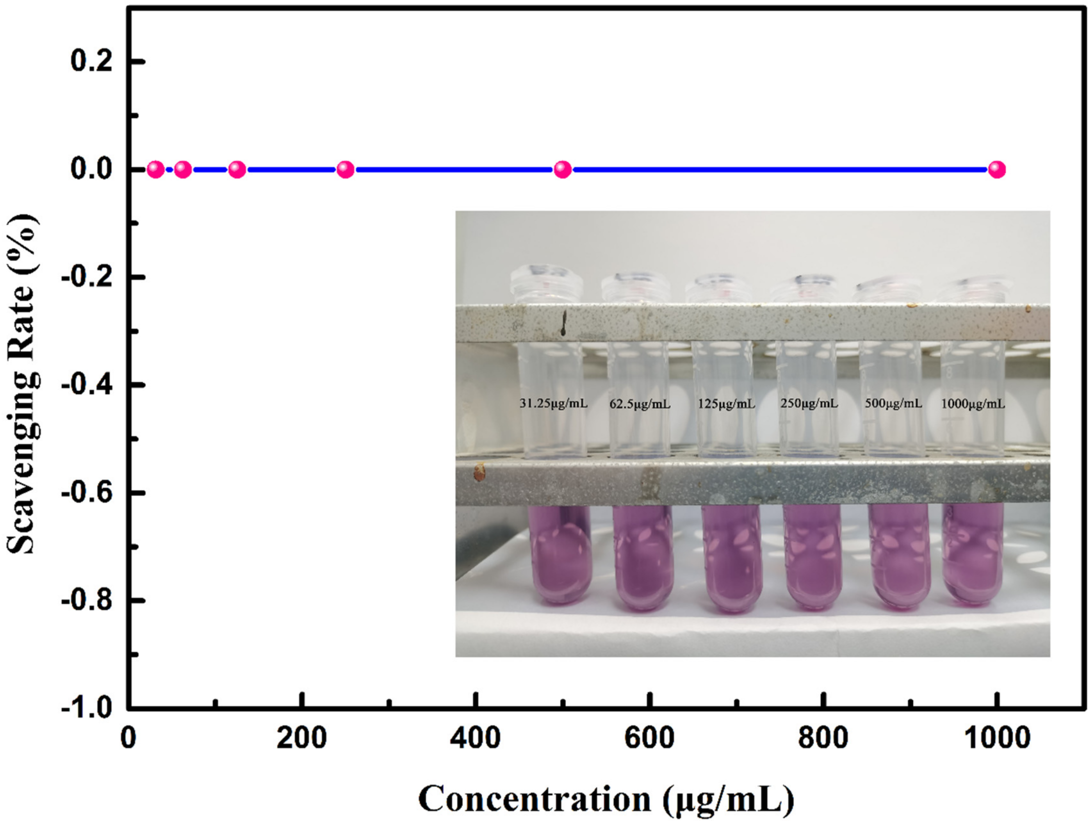

Figure S7. Antioxidant activity of pure material NMMCNs-2.

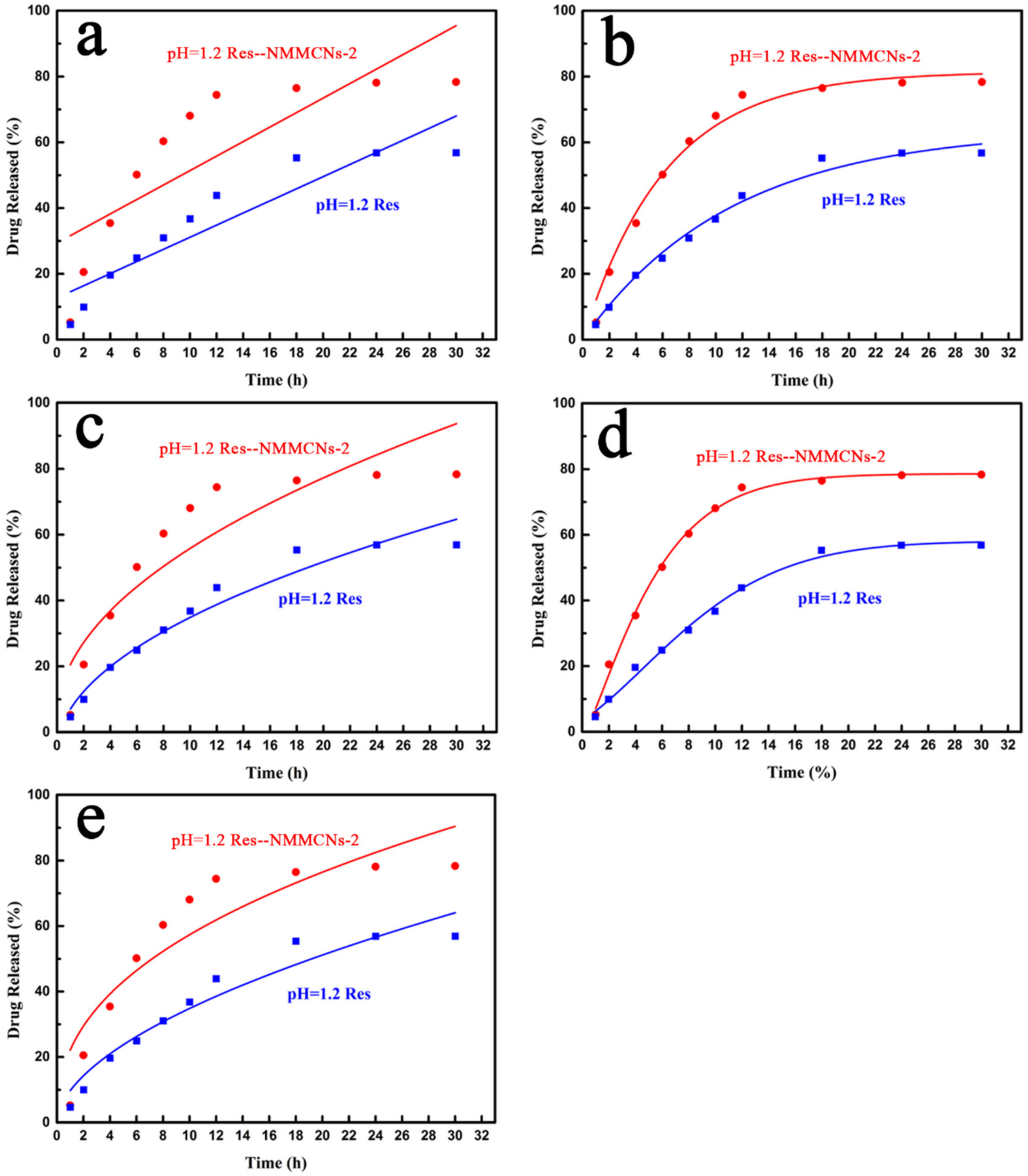

Figure S8. Fitting of release data performed at pH 1.2 for Res and Res–NMMCNs-2 to zero order (a), first order (b), Higuchi (c), Weibull (d) and Ritger–Peppas (e) models.

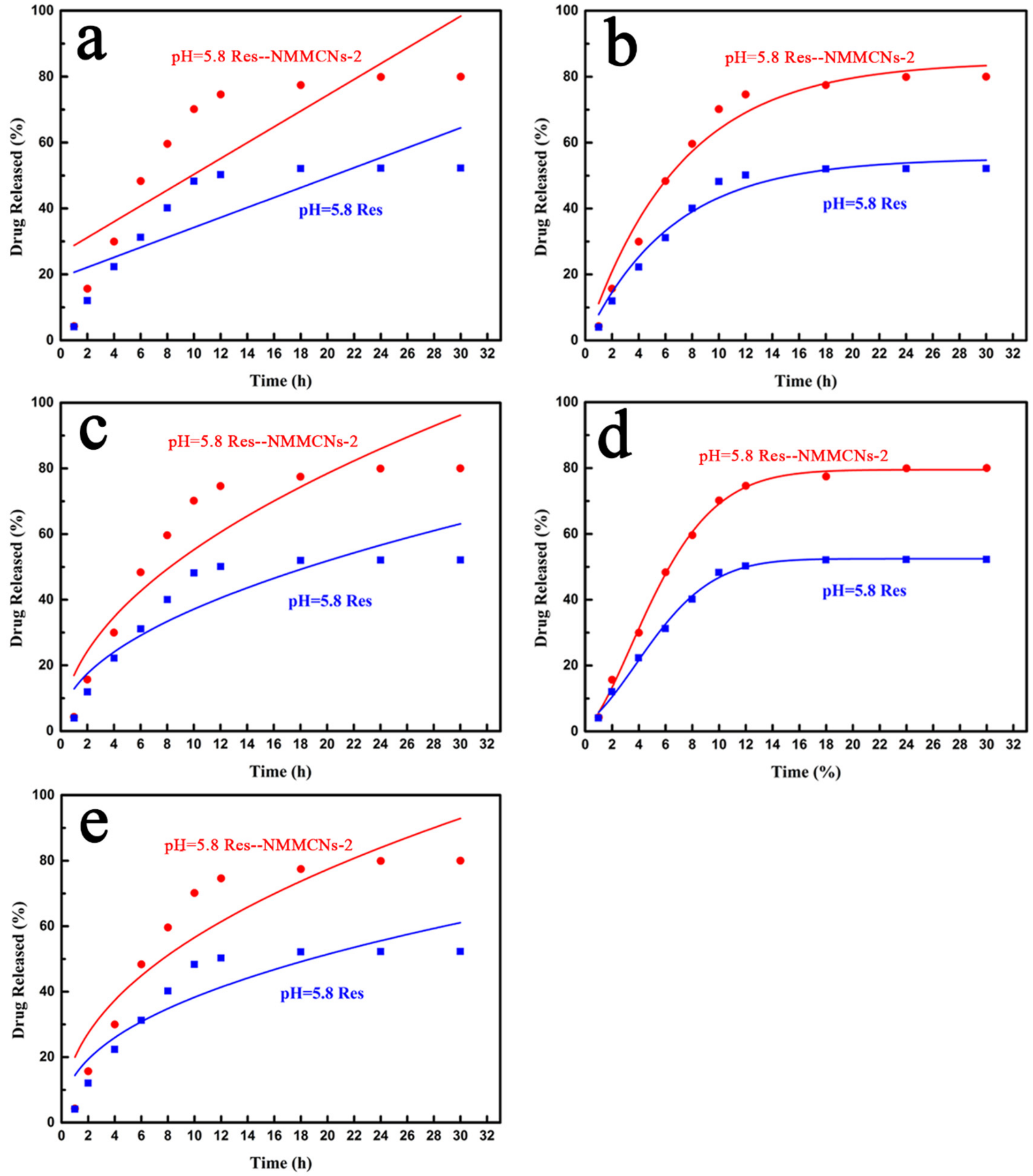

Figure S9. Fitting of release data performed at pH 5.8 for Res and Res–NMMCNs-2 to zero order (a), first order (b), Higuchi (c), Weibull (d) and Ritger–Peppas (e) models.

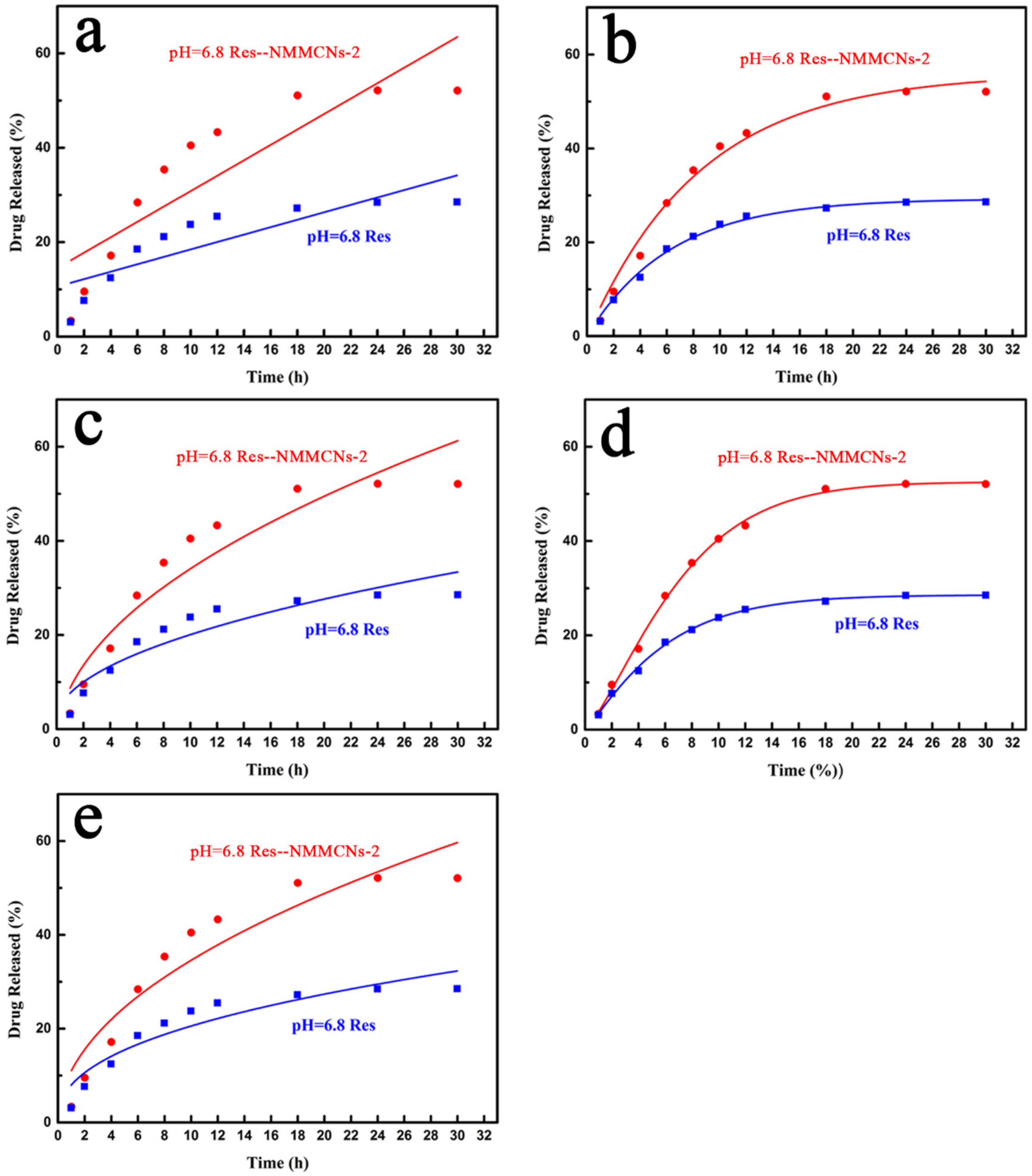

Figure S10. Fitting of release data performed at pH 6.8 for Res and Res–NMMCNs-2 to zero order (a), first order (b), Higuchi (c), Weibull (d) and Ritger–Peppas (e) models.

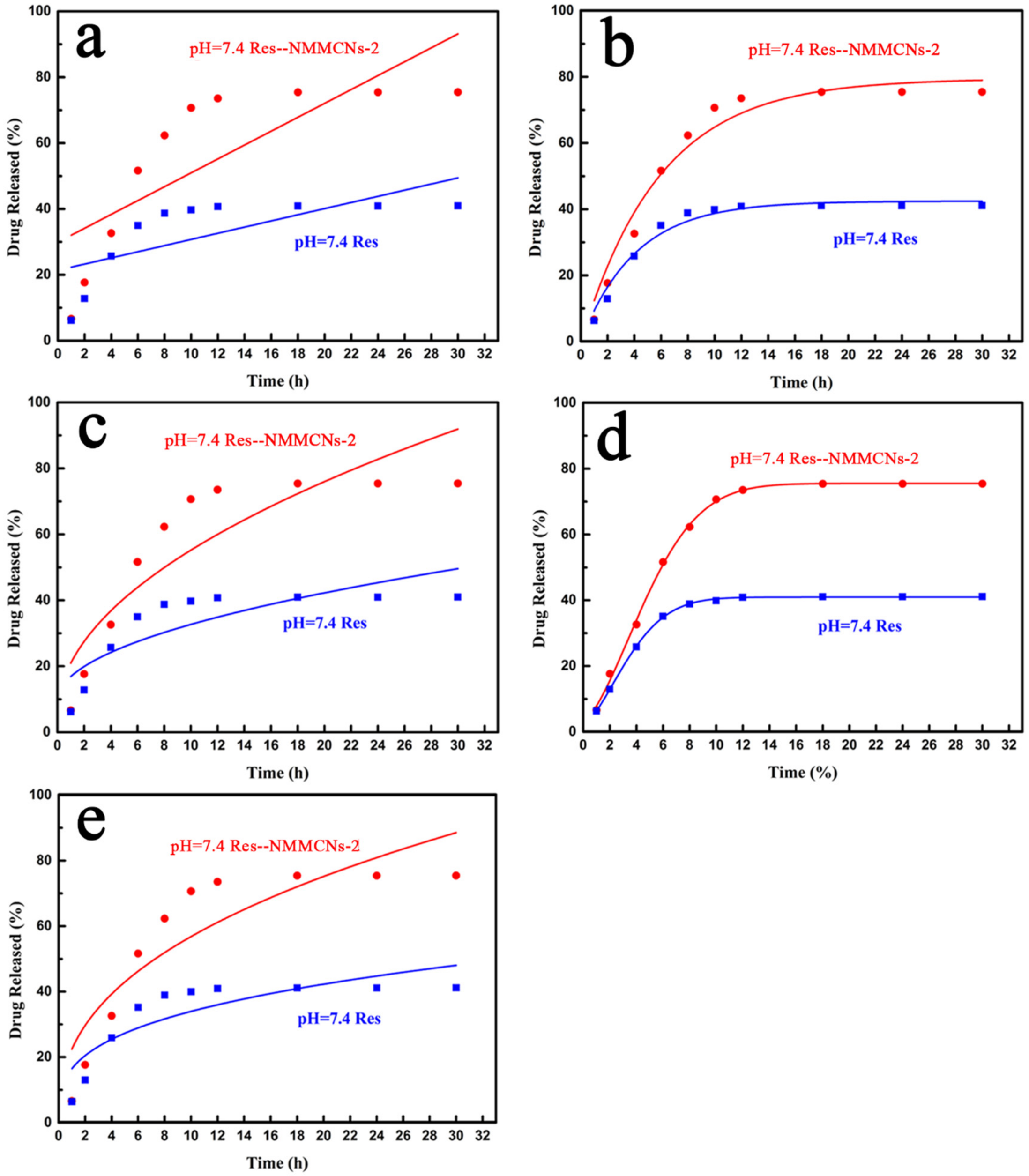

Figure S11. Fitting of release data performed at pH 7.4 for Res and Res–NMMCNs-2 to zero order (a), first order (b), Higuchi (c), Weibull (d) and Ritger–Peppas (e) models.

Table S1. Kinetic models applied to describe the release of Res from mesoporous materials.

pH Value	Equation Model	Material	Equation	R^2
1.2	Zero order	Res--NMMCNs-2	$Q = 2.20215t + 29.39683$	0.6176
		Res	$Q = 1.84367t + 12.687$	0.8384
	First order	Res--NMMCNs-2	$Q = 81.43385(1 - e^{-0.16073t})$	0.9812
		Res	$Q = 63.92634(1 - e^{-0.08917t})$	0.9878
	Higuchi	Res--NMMCNs-2	$Q = 16.34987(t^{(1/2)}) + 4.13933$	0.8121
		Res	$Q = 12.87613(t^{(1/2)}) - 5.94618$	0.9508
	Weibull	Res--NMMCNs-2	$Q = 78.57612(1 - e^{(-(0.17873(t-0.18801))^{1.22419})})$	0.9919
		Res	$Q = 57.96113(1 - e^{(-(0.08623(t+2.15403))^{1.6697})})$	0.9919
	Ritger-Peppas	Res--NMMCNs-2	$Q = 22.08293(t^{0.41451})$	0.8353
		Res	$Q = 9.66609(t^{0.5556})$	0.9385
5.8	Zero order	Res--NMMCNs-2	$Q = 2.3992t + 26.42285$	0.6363
		Res	$Q = 1.51168t + 19.06421$	0.6076
	First order	Res--NMMCNs-2	$Q = 84.56834(1 - e^{-0.14175t})$	0.9677
		Res	$Q = 55.25011(1 - e^{-0.15343t})$	0.9685
	Higuchi	Res--NMMCNs-2	$Q = 17.69784(t^{(1/2)}) - 0.73881$	0.8234
		Res	$Q = 11.22959(t^{(1/2)}) + 1.70715$	0.8005
	Weibull	Res--NMMCNs-2	$Q = 79.48219(1 - e^{(-(0.14913(t+0.38031))^{1.63135})})$	0.9970
		Res	$Q = 52.39074(1 - e^{(-(0.13048(t+1.52972))^{1.95179})})$	0.9944
	Ritger-Peppas	Res--NMMCNs-2	$Q = 19.97255(t^{0.45196})$	0.8308
		Res	$Q = 14.31836(t^{0.42615})$	0.8184
6.8	Zero order	Res--NMMCNs-2	$Q = 1.63143t + 14.52986$	0.7364
		Res	$Q = 0.78559t + 10.62437$	0.6696
	First order	Res--NMMCNs-2	$Q = 55.96257(1 - e^{-0.11704t})$	0.9838
		Res	$Q = 29.20556(1 - e^{-0.15837t})$	0.9934
	Higuchi	Res--NMMCNs-2	$Q = 11.73745(t^{(1/2)}) - 3.02131$	0.8958

		Res	$Q = 5.75713\left(t^{(1/2)}\right) + 1.84761$	0.8519
	Weibull	Res--NMMCNs-2	$Q = 52.51195(1 - e^{(-(0.13198(t+0.08189))^{1.33943})})$	0.9970
		Res	$Q = 28.54543(1 - e^{(-(0.16789(t-0.06096))^{1.1218})})$	0.9966
	Ritger-Peppas	Res--NMMCNs-2	$Q = 11.03659(t^{0.4963})$	0.8900
		Res	$Q = 7.96101(t^{0.41206})$	0.8747
	Zero order	Res--NMMCNs-2	$Q = 2.10785t + 29.88874$	0.5611
		Res	$Q = 0.93578t + 21.41698$	0.4273
	First order	Res--NMMCNs-2	$Q = 79.46824(1 - e^{-0.16895t})$	0.9655
		Res	$Q = 42.31393(1 - e^{-0.24322t})$	0.9706
7.4	Higuchi	Res--NMMCNs-2	$Q = 15.85382\left(t^{(1/2)}\right) + 5.08144$	0.7635
		Res	$Q = 7.30446\left(t^{(1/2)}\right) + 9.58045$	0.6454
	Weibull	Res--NMMCNs-2	$Q = 75.52966(1 - e^{(-(0.14931(t+1.10877))^{1.90607})})$	0.9978
		Res	$Q = 40.82445(1 - e^{(-(0.21477(t+0.67395))^{1.76387})})$	0.9995
	Ritger-Peppas	Res--NMMCNs-2	$Q = 22.43063(t^{0.40368})$	0.7920
		Res	$Q = 16.2832(t^{0.31693})$	0.7229
